# Auxin and carbohydrate control flower bud development in *Anthurium andraeanum* during early stage of sexual reproduction

**DOI:** 10.1186/s12870-024-04869-0

**Published:** 2024-03-02

**Authors:** Xiao Wan, Long-Hai Zou, Xiaoyun Pan, Yaying Ge, Liang Jin, Qunyang Cao, Jiewei Shi, Danqing Tian

**Affiliations:** 1 Zhejiang Institute of Landscape Plants and Flowers, Zhejiang Academy of Agricultural Sciences, Hangzhou, 311251 Zhejiang China; 2 State Key Laboratory of Subtropical Silviculture, Bamboo Industry Institute, Zhejiang A&F University, Hangzhou, 311300 Zhejiang China

**Keywords:** *Anthurium andraeanum*, *ARP*, Auxin, Carbohydrate, Floral bud development, *SPL12*

## Abstract

**Background:**

Flower buds of *Anthurium andraeanum* frequently cease to grow and abort during the early flowering stage, resulting in prolonged planting times and increased commercialization costs. Nevertheless, limited knowledge exists of the mechanism of flower development after initiation in *A. andraeanum*.

**Results:**

In this study, the measurement of carbohydrate flow and intensity between leaves and flowers during different growth stages showed that tender leaves are strong sinks and their concomitant flowers are weak ones. This suggested that the tender leaves compete with their concomitant flower buds for carbohydrates during the early growth stages, potentially causing the abortion of the flower buds. The analysis of transcriptomic differentially expressed genes suggested that genes related to sucrose metabolism and auxin response play an important role during flower bud development. Particularly, co-expression network analysis found that *AaSPL12* is a hub gene engaged in flower development by collaborating carbohydrate and auxin signals. Yeast Two Hybrid assays revealed that *Aa*SPL12 can interact with *Aa*ARP, a protein that serves as an indicator of dormancy. Additionally, the application of exogenous IAA and sucrose can suppress the expression of *AaARP*, augment the transcriptional abundance of *AaSPL12*, and consequently expedite flower development in *Anthurium andraeanum*.

**Conclusions:**

Collectively, our findings indicated that the combination of auxin and sugar signals could potentially suppress the repression of *Aa*ARP protein to *Aa*SPL12, thus advancing the development of flower buds in *Anthurium andraeanum*.

**Supplementary Information:**

The online version contains supplementary material available at 10.1186/s12870-024-04869-0.

## Background

*Anthurium andraeanum* which is of high commercial value occupies an important place in the world flower trade. *A. andraeanum* has multiple growing tips on its shortened stem and each growing tip produces one scape after one leaf in order. The scape is comprised of a spathe, a cylindrical spadix and a pedicel, of which the spathe is the main ornamental part. The most serious production problem of *A. andraeanum* is the excessive up-front cost caused by the time-consuming process of vegetative growth [1, 2]. Generally, this species requires a span of 2–3 years to produce the first flower after sowing. Even seedlings generated from tissue culture still undergo a vegetative-growth period of 1–1.5 years before they can bloom [3]. When the subtending leaf (in the next growing tip) is in a rapid elongation stage of the leaf petiole, flower buds in the last growing tip grow slowly and occasionally abort [4] (Figure S1). This causes a delay in the blooming of *A. andraeanum* and, consequently, a delayed selling date.

Carbon partitioning is a fundamental issue in plant biology and development process [5]. It has been found that flower/fruit abortion is mainly caused by a deficiency in or competition for carbohydrates and nutrients among growing organs [6]. In commercial production of *A. andraeanum*, removing young subtending leaves will accelerate the emergence of their concomitant flowers [4]. Moreover, a reduction in leaf area will lead to a shortage of carbon sources for follow-up inflorescences and a decrease in blooming [7, 8]. These pieces of evidence suggest that carbon source competition exists between flower buds and immature leaves in a growing tip, potentially leading to early flower abortion. These studies also imply that carbon partitioning is involved in both the timing of blooming and the number of flowers. Hence, sorting out the direction and intensity of assimilation’s flow and elucidating the working mechanism of carbohydrates during floral development would help to comprehend flower abortion during the early growth stages.

Carbohydrates have been found to regulate *SQUAMOSA PROMOTER BINDING-LIKE*s (*SPL*s), which are crucial elements associated with flowering, thereby influencing the timing, pattern and size of floral organs [9, 10]. The post-transcriptional levels of *SPL*s are negatively regulated by *miR156* which is inhibited by a high concentration of trehalose-6-phosphate (T6P) caused by high sink strength [11, 12]. The synthesis of T6P is catalyzed by trehalose 6-phosphate synthase (TPS) from glucose-6-phosphate and Urdine Diphosphate (UDP)-glucose [13]. Several studies have identified *TPS* genes in a variety of plants, such as *Oryza sativa*, *Arabidopsis thaliana*, *Populus trichocarpa*, and *Moringa oleifera* [14–17]. These genes can be divided into two subclasses: class I and class II. Class I genes have been confirmed to possess TPS enzyme activity, whereas class II genes have not been shown to encode either TPS or TPP catalytic enzymes [15, 18–21]. In *Arabidopsis*, the TPS gene family consists of 11 members (*AtTPS1*-*11*), of which *AtTPS1*–*4* belong to class I and *AtTPS5*–*11* belong to class II [14]. Notably, *AtTPS1* is a key component in the process of sugar metabolism [22]. Moreover, only *MoTPS1* and *OsTPS1* genes from *Moringa oleifera* and rice, respectively, have been found to encode TPS proteins with catalytic activity [15, 17].

SPLs mainly have a significant impact on the timing of flowering (age pathway), and the development of floral organs. The age pathway in the flowering regulation pathway is mainly determined by *miR156* [23, 24], which prolongs plant’s juvenile stage, delays flowering [25–27], reduces plant height and spike size [28]. *SPL9* and *SPL15*, represent an evolutionary branch of the Arabidopsis SPL family and have functions in flower induction and flower organ development [29–32]. *miR156*-SPL can also affect flower organ size and fruit development. Overexpression of *miR156* increases cell count, while overexpression of *SPL3*, *SPL4, SPL5*, and *SPL15* decreases cell count, controlling floral organ growth by affecting cell division [33]. Recently, *miR156*-SPL has been shown to affect flower organ size and ovule formation by regulating MADS-box and auxin signaling. Overexpression of *LeMIR156b* precursors in tomato did not reduce the volume of floral organs, but significantly affected fruit development [34], suggesting that *miR156*-SPL has different effects on reproductive organs in different species. SPLs is linked to fertility as well. Increased expression of *GhSPL*, the target gene of *miR157*, can activate the transcription of MADS-box genes in cotton, such as *AGL6* and *SITDR8*. MADS-box transcription factors or unknown factors may bind to auxin-responsive motifs in the promoters of downstream genes to influence auxin signaling factors and further ensure the normal production of organ primordials (such as ovule) and promote cell proliferation and cell amplification [35]. Similarly, in *Arabidopsis thaliana*, the insertion of transposons leads to a loss of *SPL8* gene function, resulting in decreased fertility, mainly due to abnormal cell differentiation during anther formation. In addition, knockout of the *SPL8* gene affects the formation of glandular hairs in megaspore and sepal, as well as the lengthening of stamen filaments [36, 37]. Interestingly, SPLs can also interact with proteins and be involved in the regulation of flower development. For example, *At*SPL10 can physically interact with *At*WRKY12/13 to regulate the target gene *miR172b* in the mediation of flowering [38].

Polar auxin transport (PAT), auxin content and auxin transduction are closely associated with floral meristem initiation and development, floral organ initiation and boundary establishment, floral organ outgrowth, floral meristem determinacy, gynoecium development, and ovule development. Auxin efflux regulator *PIN* encodes a transmembrane transport protein for auxin [39]. *PINOID* encodes a serine/threonine protein kinase involved in the polar transport of auxin. The *pin1* and *pinoid* mutants showed defects in flower development [40] and multiple defects in inflorescence development, indicating that auxin is necessary for the initiation of flower primordia. The *DR5*::*GUS* gene (Auxin response element fused to β-glucuronidase) can be used to represent the level of free auxin [41]. Aloni et al. [42] showed that the removal of any flower organ led to an increase in *DR5*::*GUS* expression and inhibited the vertical development of other flower organs. This could be due to the significant amount of auxin at the site of organ removal, which inhibits the vertical growth of other flower organs. Auxin signal transduction is also crucial for flower development. Auxin response factor (ARF) plays a central role in auxin signal transduction by binding to auxin response elements to either promote or inhibit gene expression associated to this biological process. For example, the flowering time extension in *arf6* and *arf8* mutants was suppressed during flower opening, indicating that *ARF6* and *ARF8* positively regulate flower organ differentiation [43]. In addition, *LEAFY51-52*, *SEU54* and many other genes also affect the formation of flower organs by influencing auxin signal transduction [44–47].

The present study aims to analyze the nutrient distribution process during the early stages of sexual reproduction in *A. andraeanum*, in order to comprehend the rationale behind the abortion of flower buds. Firstly, we explored the source-sink relationship between flowers and leaves during different development periods. Secondly, we used transcriptomes to parse the gene regulatory network during flower development. Thirdly, the Yeast Two Hybrid (Y2H) system was constructed to screen for proteins that interact with *Aa*SPL12. Finally, in a trial with spraying a certain concentration of sucrose and IAA on *A. andraeanum*, we measured the effect of sucrose on flowering and the expression of both *AaARP* and *AaSPL12*. The potential functions of these two genes in the flower development of *A. andraeanum* are also discussed in detail. Our findings demonstrate the connection between nutrient accumulation and internal signals during the development of *A. andraeanum*.

## Results

### Growth stages of Anthurium andraeanum

**Fig. 1:**
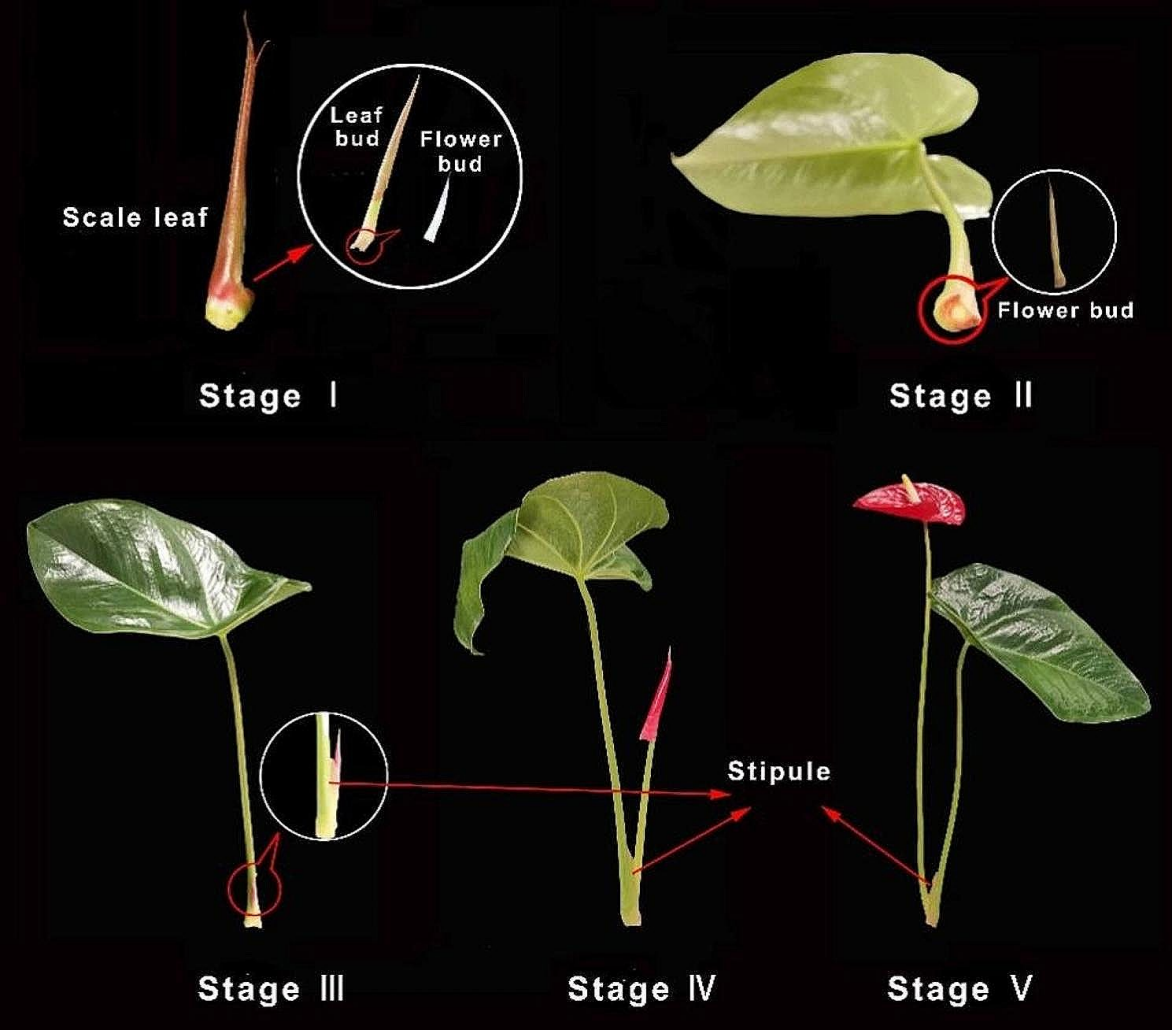
Flower growth stages of *Anthurium andraeanum*. Stage I: both leaf bud and flower bud are present but contained in a scale leaf. The leaf bud and flower bud are shown under magnification in a white circle. Stage II: the flower bud is incubated in the petiole of its concomitant leaf which protrudes out from the scale leaf. Stage III: the flower bud protrudes from the petiole of its concomitant leaf. Both stipule and flower bud are shown under magnification in a white circle. Stage IV: a folded spathe with a pedicel that is longer than 1/2 the length of its concomitant petiole. Stage V: the spathe is fully bloomed

According to the growth form, the flower growth process of *A*. *andraeanum* was distinguished into five stages artificially (Fig. 1). Stage I is defined as a period when a leaf bud and a white flower bud are embedded in a scale leaf. When the flower growth switches to stage II, the leaf bud protrudes from the scale leaf until it fully unfolds and the flower bud remains embedded in the base of the petiole. During stage III, the flower bud emerges from the stipule in the petiole base of its concomitant leaf. In the meantime, the concomitant leaf is broadening and thickening. Stage IV is characterized by a curly spathe with a pedicel that is longer than 1/2 the length of its concomitant petiole. During this stage, the bract is enlarging and the concomitant leaf is still thickening and becoming waxy. When the spathe is unfolded, the flower growth is considered to have entered stage V which is the optimum viewing time.

### Source-sink status between flowers and their concomitant leaves

**Fig. 2:**
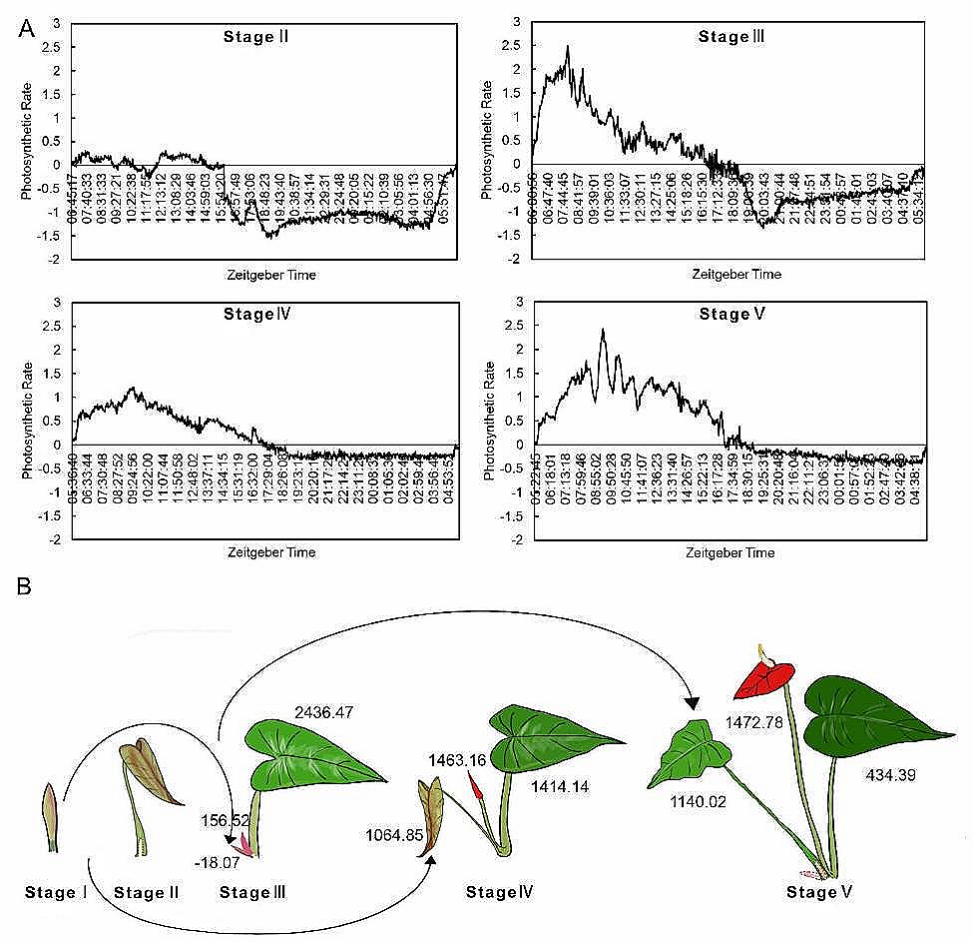
Source-sink relationship between flowers and leaves in different development periods of *Anthurium andraeanum*. (A) Photosynthetic rates of the concomitant leaf form stage II to stage V. (B) Isotope labeling of the newborn flower, the concomitant leaf and the subtending leaf from the next round of growth point at different stages; Numbers in the diagram represent ^13^C abundance in the corresponding parts

To ascertain the existence of a source-sink relationship between flowers and their accompanying leaves, we measured the photosynthetic rates of leaves at various stages (Fig. 2A). Due to being embedded in the concomitant scale leaf, the leaf buds at stage I were not measured for photosynthetic measurement. The continuous 24-hour photosynthetic rates showed that leaves at stage II had slightly positive photosynthesis during the daytime and then respired strongly at night, indicating that the newborn leaves were in a sink state. In contrast to respiration-based consumption, net photosynthetic accumulation was observed in the concomitant leaves at stages III, IV and V. It should be noted that the highest carbon accumulation was found at stage V, the intermediate one at stage IV, and the lowest one at stage III.

We further employed carbon isotope labelling to detect the direction and intensity of photosynthetic flow among the newborn flower, the concomitant leaf and the subtending leaf from the next round of growth tip (Fig. 2B; Figure S2). The ^13^C abundance of the concomitant leaf was highest (2436.47) at stage III. In the meantime, low abundances were detected in the concomitant flower bud and the subtending leaf bud (156.52) from the next round of growth point (-18.07, at stage I). These results suggest that the concomitant leaf is a strong sink at stage III. At stage IV, the ^13^C abundances of the concomitant leaves decreased to 1414.14, while those of the concomitant flowers and the subtending leaves from the next round of growth point (at stage II) rose to 1463.16 and 1064.85, respectively. This suggests that the concomitant leaves serve as a source, while the latter two serve as sinks. It is very clear that the flower and the subtending leaf from the next round of growth points must compete for carbon flow from the concomitant leaf. In particular, the ^13^C abundances of the fully bloomed flower and the subtending leaf from the next growing point (usually in stages II–III) were much higher than those contained in the concomitant leaves. These data indicate that there is source-sink competition between the newborn flower and the subtending leaf from the next growing point, as well as source-sink conversion in the concomitant leaf during flower growth.

### TPS activity and *TPS* expression level

**Fig. 3:**
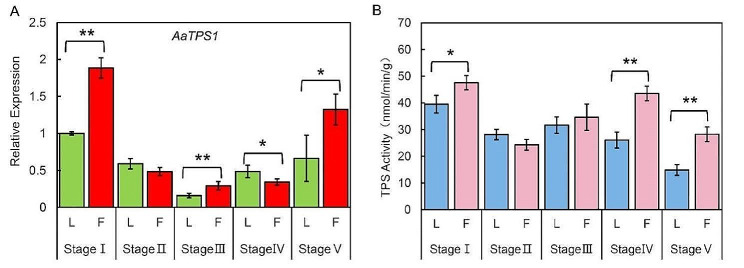
*TPS* expression and TPS enzyme activity between the flowers and the concomitant leaves during stage I to stage V. (A) *TPS1* expression level in leaves and the corresponding flowers during different developmental periods. (B) TPS activity of leaves and the corresponding flowers during different developmental periods. Three biological replicates were tested in the experiments. “*” indicates that the difference in *TPS1* expression level or TPS activity is significant (*P*<0.05). “L” represents leaves; “F” represents flowers

TPS activity was a detector for sucrose allocation between the leaf and flower [48]. Herein, we examine *TPS1* gene expression and TPS activity among the newborn flowers and their concomitant leaves at the five stages. The results indicated that 49 sequences containing Glyco_transf_20 and Trehalose-PPase domains were identified, eight of which were clustered with the *Arabidopsis* class I TPS genes (*AtTPS1*–*4*) (Figure S3). Sequence alignment analysis indicated that these eight isoforms are derived from the same gene. Therefore, we designed amplification primers in the shared sequence section (Supplementary 5). The expression level of *TPS1* was significantly higher in flowers than in leaves at stages I, III, and V, but the reverse applies at stage IV (Fig. 3A). TPS activity in the newborn flower was significantly higher than that in the concomitant leaves at stages I, IV, and V (Fig. 3B). The overall TPS activity and its gene expression level at stage I were higher than those at other stages. These results suggest that both the leaf and flower at early growth stages are in a sink state.

### Auxin metabolic pathway involved in flower bud development

**Fig. 4:**
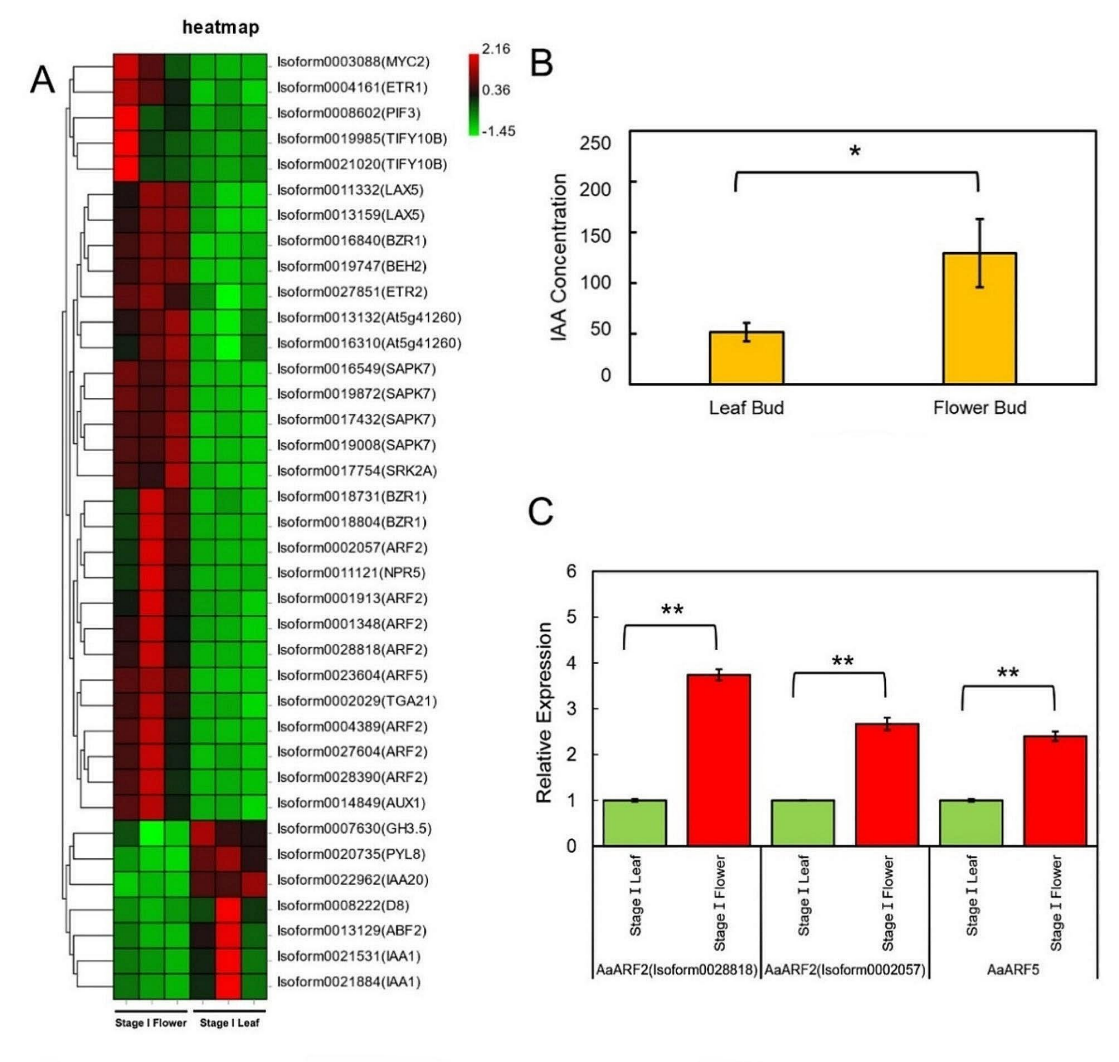
Gene expression profiles associated with auxin regulatory pathways and IAA content in different tissues. (A) Gene expression heatmap of hormone metabolic pathway in flower buds and leaf buds at Stage I. (B) Auxin concentration of flower buds and leaf buds at Stage I. (C) *AaARF2*’s and *AaARF5*’s expression levels in both flower buds and leaf buds at Stage I. Three biological replicates were tested in the experiments. The data are presented as means ± SEM. “*” indicates statistical differences between leaves and flowers (*P*<0.05)

A total of 29.99 gigabytes (GB) of raw data were obtained from the two libraries (0–4 and 4–10 kb libraries) through PacBio *Iso-Seq* sequencing. A total of 29,189 unique full-length transcripts (isoforms) were obtained through pipeline analysis. Moreover, 1.796 billion pairs of clean reads were produced from 30 libraries using RNA-Seq to calculate the expression level (fragments per kilobase per million, FPKM) of each isoform, with a mapping ratio of 77.20–85.39%. Auxin metabolism has been reported to be associated with carbohydrate partition during floral development [49, 50]. To investigate whether there is a relationship between auxin and sugar in the anthesis of *A. andraeanum*, the auxin metabolic pathway was analyzed in this study. The isoforms involved in the hormone metabolic pathway were screened from the transcriptome data set, and heatmap analysis was used to illustrate the expression profiles of genes in stage I based on their FPKM values. The hormone metabolic pathways involved in auxin (*LAX5*, *ARF2*, *ARF5*, *TGA21*, *AUX1*, *GH3.5*, *IAA20*, and *IAA1*), jasmonic acid (*MYC2* and *TIFY10B*), ethylene (*ETR1* and *ETR2*), brassinolide (*BZR1*, *BEH2*, and At5g41260), abscisic acid (*PIF3*, *SAPK7*, *SRK2A*, *PYL8*, and *ABF2*), salicylic acid (*NPR5* and *TGA21*) and gibberellic acid (*PIF3* and *D8*) signaling pathway were depicted in Fig. 4A. Among these thirty-seven genes, seven were upregulated in leaves, while the remaining thirty were upregulated in flower buds. Auxin signaling way involves fifteen genes, eleven of which (eight auxin response factors: *ARF*s; two auxin transfer genes: *LAX*s; one auxin inhibitor: *AUX1*) [51–53] were expressed at higher levels in flower buds and four of which (three auxin inhibitors: *IAA*s; one auxin steady state regulator: *GH3.5*) [51, 54] were expressed at lower levels (Fig. 4A). The heatmap indicated that the genes related to auxin in flower buds had a high transcription abundance at stage I. To further verify the results in Fig. 4A, we then measured the concentration of IAA in flower buds at stage I and the expression levels of auxin responsive related genes (*AaARF2* and *AaARF5*) (Fig. 4B, C). The IAA concentration in flower buds was more than twice as high as in leaf buds. The CDS sequence information of *ARF2* suggests that the seven isoforms of *ARF2* are associated with two genes, thus two representative isoforms (isoform0028818 and isoform0002057) were chosen for gene expression analysis (Supplementary 5). The expression of *AaARF2* (isoform0028818 and isoform0002057) and *AaARF5* in flower buds were 3.7, 2.7, and 2.4 times higher respectively than in leaf buds. These results suggest that auxin plays a significant role in the early stages of flower development.

### Auxin-related genes were co-expressed with *Aa*SPL12

**Fig. 5:**
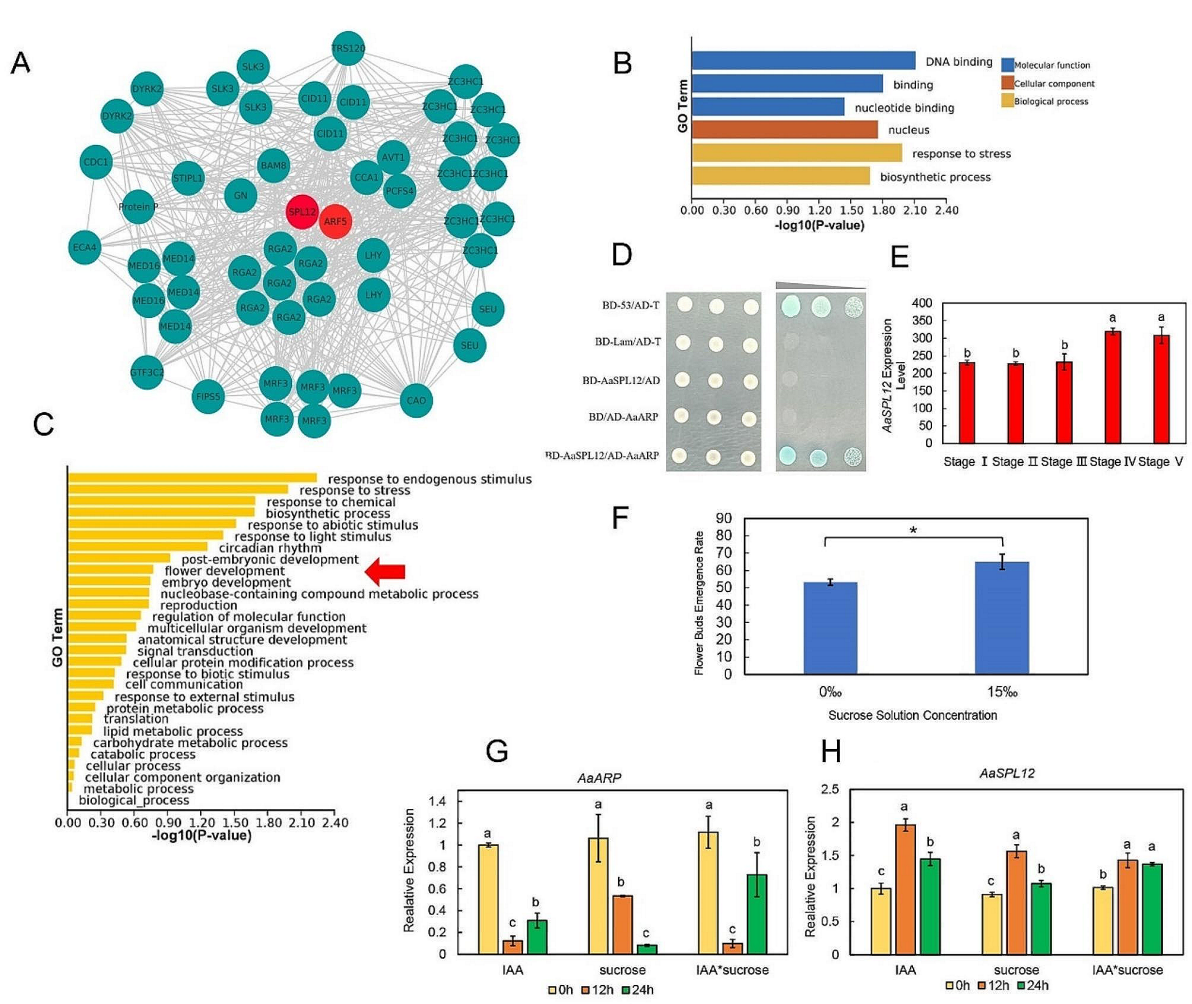
*AaSPL12* is a positive regulator in floral development of *Anthurium andraeanum*. (A) Gene module based on *AaSPL12* and *AaARF5* by the analysis of ClusterONE. (B) Enriched GO terms related to *AaSPL12* and *AaARF5* kernel gene module. (C) Biological process enrichment related to *AaSPL12* and *AaARF5* gene module. Red arrow is pointing to flower development item. (D) The Y2H assay of *Aa*SPL12 and *Aa*ARP. Dilution series of yeast Y2H Gold transformed with *Aa*SPL12 and *Aa*ARP grows on -Leu/ -Trp/ -His/ -Ade/ + X-α-gal. (E) *AaSPL12* expression level (FPKM) in different flower development stages according to the transcriptomic data. (F) External application of 15‰ (w/v) sucrose promotes the flowering rate. (G) Expression level of *AaARP* under IAA and sucrose treatment. (H) Expression level of *AaSPL12* under IAA and sucrose treatment. Data are presented as means ± SEM. The different parts effect was evaluated by ANOVA one-way followed by a LSD *post hoc* analysis. *P*<0.05 was considered significant

To explore the relationship between sucrose and auxin, co-expression network analysis was performed based on the transcriptomes of the flowers and their concomitant leaves. The co-expression network analysis indicated that *AaSPL12* has strong co-expression relationships with the transcription factor *AaARF5* and other genes involved in plant development, flower development (*e.g. AaLHY*, *AaRGA2*, *AaMRF3*, and *AaSEU*), cell division (*e.g. AaSLK3*), carbohydrate metabolism (*e.g. AaBAM8*), and auxin polar transport (e.g. *AaGN*) (Fig. 5A). Gene functional modules of enrichment related to *AaSPL12* and *AaARF5* indicated that the biological processes include floral development and embryo development items (Fig. 5B, C). Moreover, *AaSPL12* exhibits a persistent increase in expression in the flower tissue (spathe) during the flowering phase (Fig. 5E), implying that it is likely involved in the flower development process. Consequently, we propose that *AaSPL12* may serve as a hub gene that combines the auxin metabolic pathway and the carbohydrate metabolism pathway.

### Protein-protein interaction between *Aa*SPL12 and *Aa*ARP

To investigate the interaction between *Aa*SPL12 and other proteins, a yeast two-hybrid screen was carried out. All positive clones were scratched off for NGS (Next Generation Sequencing) using Illumina NovaSeq 6000 system. Of the pGADT7-*Anthurium* cDNA, an auxin-repressed protein (*Aa*ARP) was clearly detected for pGBKT7-*Aa*SPL12. We then conducted a point-to-point assay to verify the NGS result (Fig. 5D). Our results indicated *Aa*SPL12 can interact with *Aa*ARP, a gene that has been proved to be completely repressed by both auxin and sucrose [55].

When we applied a 15‰ sucrose solution externally to leaves of non-flowering plants of *A. andraeanum*, we observed a considerable effect on the flowering rate (Fig. 5F). We further treated *Anthurium andraeanum* with IAA and sucrose solution to verify the responsiveness of *AaARP* and *AaSPL12*. The results showed that *AaARP* was strongly repressed by IAA, sucrose and IAA + sucrose treatments at 12 h, respectively (Fig. 5G, H). The expression of *AaARP* was higher at 24 h under both IAA and IAA + sucrose treatments compared to 12 h; however, the expression level under sucrose treatment decreased drastically at 24 h when compared to 12 h. The duration of the inhibitory action of sucrose on *AaARP* surpasses that of IAA treatment. The expression of *AaSPL12* was significantly up-regulated under all three types of treatments (Fig. 5H). Nevertheless, the expression of *AaSPL12* decreased under IAA and sucrose treatment at 24 h.

## Discussion

### Sucrose functions in *Anthurium andraeanum*’s flower bud development

*Anthurium andraeanum* is a plant with a continuous flowering habit, where each leaf is connected to one flower, a characteristic that has been linked to a constant cycle of source and sink switching [7]. In this study, we uncovered the sink-source status of the newborn flower and its concomitant leaf at different growth stages (Fig. 2). Both the leaf and flower were in a weak sink status at stages I and II and the leaf was in a weak source status, while the corresponding flower was in a weak sink status at stage III. These findings were supported by the photosynthetic measurements and the records of carbon flow using isotope labeling (Fig. 2).

The alteration cycle of sources and sinks in *A. andraeanum* suggests that sucrose partition is closely related to different development stages. Trehalose-6-phosphate (T6P) is a chemical intermediate in the sucrose metabolic pathway and trehalose synthesis pathway [56]. It can be synthesized by TPS, which catalyzes the production of glucose uridine diphosphate (UDPG) and glucose-6-phosphate (G6P), and is then converted into trehalose by trehalose-6-phosphate phosphatase (TPP) [13]. Numerous pieces of evidence have proven that T6P is closely positive on to the intensity of sucrose sinks and is unrelated to other sugars; T6P plays a dual role in coding sucrose supply as both a steady state and signaling entity [57, 58]. In the source leaves, T6P adjusts the production of sucrose to balance the supply and growing demand for sucrose. Meanwhile, as a signal of availability, T6P could link the development of source organs to sucrose supply and influence developmental decisions, such as blooming, embryogenesis, and branching, which represent the future requirements for sucrose. Before the first flower comes out, *A. andraeanum* keeps on a “pseudo vegetative growth” but along with abortive flower buds covered by the stipules. Once one flower comes out successfully, the whole plant steps into the “one leaf to one flower” cycle. The TPS activity and gene expression level of *AaTPS1* in successfully flowering turn indicated that the flower buds in stage I was in a strong sink state and needed sufficient sucrose. For developmental transitions to take place, a sufficient amount of carbon source is required; however, an imbalance in its distribution can lead to flower bud abortion [59]. Nevertheless, at stage IV of the species, the expression level of the *AaTPS1* gene and the TPS activity showed a disparity between flowers and leaves (Fig. 3). On one hand, the accelerated growth of leaves with photosynthetic abilities may be attributed to their dual roles as both strong sinks and strong sources. Hence, a correlation exists between high sucrose concentration and high TPS activity. On the other hand, a time gap may exist between gene expression and the related protein translation. The *AaTPS1* gene in stage IV leaves may be temporarily overexpressed due to external or light induced factors, though it rapidly decreases after a short-term stress expression and the protein may maintain a relatively long-term high concentration.

### Hormones affect flower bud development in *Anthurium andraeanum*

An analysis of the hormone metabolic pathway using a gene expression heatmap revealed that the early stages of sexual reproduction involve auxin, jasmonic acid, ethylene, brassinolide, abscisic acid, salicylic acid, and gibberellic acid signaling pathways. However, the expression levels of cytokinin metabolic pathway genes did not show a significant difference between flower and leaf buds in Stage I. The similarity in the expression of cytokinin genes between flower buds and leaf buds, both of which are in the stage of rapid cell division, may explain the lack of difference in cytokinin metabolism pathways between them. Studies have shown that spraying gibberellic acid has a positive effect on the flowering of orchids [60] and *A. andraeanum* [61]. Analysis of the hormone metabolism pathways in the transcriptome revealed that the expression of the phytochrome interacting factor PIF3 [62] was slightly higher in flower buds than in leaf buds, while the expression of the negative regulatory factor D8 [62] of the gibberellin pathway was lower in flower buds (Fig. 4A). This suggests that gibberellin may have a synergistic effect with light signals to promote the development of flower buds in *A. andraeanum*. Auxin plays an important role in flower development [53, 63, 64]. Moreover, auxin metabolism is an integral part of the regulation of sugar metabolism and transport in buds, flowers and fruits, facilitating the growth of the plant. Sucrose availability up-regulated early auxin synthesis genes (*RhTAR1* and *RhYUC1*) and the auxin efflux carrier gene (*RhPIN1*) during bud outgrowth in *Rosa hybrida* [49]. The *Rh*ARF7-*Rh*SUC2 module is involved in the process of petal abscission by mediating the interaction of auxin and sucrose [50]. T6P promotes seed filling by activating auxin biosynthesis, suggesting that the cooperation of sugar and auxin also positively occurs in fruit grouting stage [12]. In *Anthurium andraeanum*, the concentration of IAA and the expression levels of auxin responsive genes are higher in flower buds than in the corresponding leaf buds during the reproductive period (Fig. 4A, B, and C).

### *Aa*SPL12-*Aa*ARP module might engage in flower bud development

Transcriptome profiles revealed that auxin genes and sucrose related genes were crosstalk in a gene module. In particular, *Aa*SPL12 was strongly co-expressed with auxin signaling related genes *AaARF5* and genes related to flower development (*AaLHY*, *AaRGA2*, *AaMRF3*, and *AaSEU*; Fig. 5A, B, and C). Hence, *Aa*SPL12 was supposed to be the connection between auxin and sucrose in regulating flower development. Interestingly, *Aa*ARP was screened as the interactive protein of *Aa*SPL12 through the Y2H system (Fig. 5D). ARP is closely related to DRM (Dormancy Associated Protein) gene, and together they form the ARP/DRM gene family [65]. ARP/DRM is often used as a dormancy marker, due to its high expression in dormant buds [66]. Pea *DRM1*, Arabidopsis *DRM1* and *ARP1*, and sorghum *DRM1* were also highly expressed in dormant axillary buds [65, 67, 68]. In particular, *ARP* is negatively expressed during the auxin-mediated elongation process [55, 69, 70] and the overexpression of *BrARP1* and *BrDRM1* genes can stop the growth of *Brassica rapa* by inhibiting cell elongation or expansion [71]. There is evidence that DRM1/ARP family members are intrinsically disordered proteins (IDPs) [72]. Most sequences of IDPs do not produce a fixed tertiary structure, so they can partially fold in response to changes in the environment resulting from physiological conditions or interactions with binding partners, providing corresponding biological functions [73]. Due to this characteristic, IDPs are often used as important components of protein interaction networks, such as LEA, GRAS, and HSP proteins [74–79]. Dormancy and germination are physiological processes that require the perception of external and physiological changes, so ARP/DRM is likely to play an important role in dormancy or breaking dormancy mechanism. To date, *ARP* has been studied more in axillary buds, branching, outgrowth of leaf buds and shoot tips, but less in flower bud development [65, 68, 80–82]. The simultaneous effect of auxin and sucrose can completely repress the expression of *ARP* [55]. In this study, qRT-PCR results showed that the transcriptional abundance of *AaARP* was strongly inhibited, while that of *AaSPL12* was significantly increased after sucrose, IAA and IAA + sucrose treatments, respectively (Fig. 5G, H). These results suggest that *AaARP* is down-regulated in auxin-sugar mediated floral bud development in *A. andraeanum*. Moreover, we found that the external application of 15‰ sucrose on the leaves of young seedlings of *A. andraeanum* significantly promote the bloom rate (Fig. 5F). The application of sucrose to leaves can effectively reduce the strength of the leaf sink, transport more carbohydrates to the flower buds, stimulate their growth and consequently enhance the flowering rate.

## Conclusions

In the present study, we conducted photosynthesis measurements, carbon isotope labelling, and TPS enzyme activity measurements to investigate the competition between flower buds and leaves during the early stages of sexual reproduction in *A. andraeanum*. The external application of sucrose was found to effectively increase the flowering rate of the plants, suggesting that carbon source is a major factor in the flowering of this species. Transcriptomic analysis revealed that the auxin metabolism pathway and its related response transcription factors are involved in the early development of flower buds in *A. andraeanum*. Co-expression network analysis demonstrated that the flowering hub gene *AaSPL12* is closely associated with auxin metabolism and sucrose metabolism. Additionally, Y2H assays identified a protein interaction between *Aa*SPL12 and the dormancy indicator protein *Aa*ARP, which is inhibited by both sucrose and exogenous auxin. Therefore, we conclude that both auxin and sucrose are involved in the growth and development of flower buds, and hypothesize that auxin and sucrose release the dormant protein *Aa*ARP from inhibiting the key flowering protein *Aa*SPL12, thus promoting flowering in *A. andraeanum*. However, further research involving transgenic plants and protein interaction validation in vivo is necessary to verify this hypothesis.

## Materials and methods

### Biological material

*A. andraeanum* cv. ‘Alabama’ plants were grown in an artificial climate chamber maintained under 12 h light (light intensity 15,000 lx, 25 ℃, 80% relative humidity) / 12 h dark (21 ℃, 80% relative humidity) cycles.

### Morphological classification of flower growth process

Flower growth processes were investigated in twenty individuals of *A. andraeanum* cv. ‘Alabama’. According to morphological characteristics of flowers and their concomitant leaves. Several flower growth stages were proposed.

### Photosynthetic measurements

The continuous 24 h photosynthetic characteristics of each leaf were determined by Li-6400XT Photosynthesis system (LI6400 Inc., Lincoln, NE, USA). The photosynthetic rate values were collected once every 1.8 min and produced 800 pieces of data. The parameters for Li-6400XT were set as follows: Leaf Fan, 3 V; Flow, 400 μm s^− 1^; Mixer, OFF; BlkT, 25 ℃; Track, Parout. The leaf photosynthetic capacity was valued by $\sum Photosynthetic rate$.

### ^13^C - labelling

Labeling experiments were conducted using carbon-13-labeled CO_2_. Leaves of *A. andraeanum* in different flower growth stages were treated with ^13^CO_2_ gas. An eight-liter (the actual volume is 6-liter after being sealed) transparent plastic bag was used to tightly cover the leaf at 07:00. To prevent injuring the leaf petiole and gas leakage, a wad of cotton was used for sealing. An air-tight glass injector was used to add 20 mL ^13^CO_2_ into the plastic bag. The pinholes were blocked off by transparent adhesive tape. The plastic bag air was shaken up after the ^13^CO_2_ injection. The bags were unfastened at 18:00. This treatment was repeated for three consecutive days.

After 48 h of standing, the treated leaves and corresponding spathe were collected. Plants without any treatment were used as the control group. The samples were pre-dried to a constant weight, ground into powder, and filtered by an 80-mesh screen. Stable isotope ratio mass spectrometry (Thermo Fisher Scientific, Waltham, MA, USA) was used to analyze the CO_2_ content. Carbon isotope composition (δ^13^C) was calculated as a deviation of the carbon isotope ratio (^13^C/^12^C called R) from the international standard: δ_13_C=(R_sample_/R_standard_ − 1) ×1000. And the total carbon content [C (%)] was calculated as: C (%)=$\frac{20}{(Wsample/Wstandard)\times (BAsample/BAstandard)}$.

### Determination of TPS activity

TPS (Trehalose-6-phosphate synthase) activity of both the leaves and their concomitant spathes from Stages I to V was determined using the Trehalose-6-phosphate-Synthase Determination Kit (Cominbio, China, cat. No.TPS-1-Y) and Synergy^TM^H1 (BioTek Ltd, USA) following the manufacturer’s description. Three biological replicates were performed at each stage.

### IAA and sucrose treatment

For the IAA and sucrose treatment experiments, eight-month-old tissue-cultured plantlets were sprayed evenly with a 100 µmol/L IAA solution, a 15‰ (w/v) sucrose solution and a 100 µmol/L IAA + 15‰ (w/v) sucrose solution, respectively, on the leaves. Leaves were collected after 0 h, 12 h, and 24 h treatment, respectively. Ten-month-old tissue cultured plantlets were sprayed with 0‰ (w/v) and 15‰ (w/v) sucrose solution. The sucrose solution was applied twice per week. The experiment was conducted three times, with sixty plants allocated to each treatment. On the 60th day after the sucrose spraying treatment, the flowering rate was recorded.

### Quantitative real–time PCR

RNA was isolated with the RNA Easy Fast Kit (DP452, TIANGEN Co. Ltd., Beijing, China) according to the manufacturer’s protocols. A 2100 Bioanalyzer (Agilent Technologies, CA, USA) was used to detect the integrity of RNA.

Primers used for qRT-PCR were listed in Table S1. qRT-PCR experiments were performed using the StepOnePlus Real-Time PCR System (ThermoFisher Scientific, USA) with SYBR® Green Realtime PCR Master Mix (Toyobo Bio. Inc). Each experimental treatment was performed in four replicates. The relative expression level was calculated as 2-ΔΔCt and normalized using 18 S as the internal standard.

### RNA sequencing and co-expression network analysis

Spathes without inflorescences and leaves without petioles were sampled from the five stages (Fig. 1), respectively, with three biological replicates. Total RNA was extracted as mentioned above. After RNA quality control as described in Wan et al. [83], the RNA samples were used for RNA-seq library construction of next generation sequencing on an Illumina HiSeq™ 4000 platform following the procedure of Zou et al. [84]. In addition, a mixture containing the RNA samples from all five stages was employed for PacBio ISO-Seq to construct a reference transcriptome. The procedures for ISO-seq library preparation, sequencing and data analysis were performed according to a previous study [85]. Data filtering and gene quantification for next generation sequencing data were performed as described by Zou et al. [84] with the reference transcriptome from ISO-seq.

Gene expression matrices from the all of bract samples were used to calculate the weighted Pearson correlation coefficient (wPCC) and mutual rank (MR) as described by Obayashi et al. [86]. ClusterONE v1.040 was used to decompose the co-expression network based on wPCC into multiple gene modules. The following parameters were employed in the ClusterONE analysis: minimum size 3, edge weight, node penalty 2, merging method single-pass, similarity match coefficient, overlap threshold 0.75, and seeding method from unused nodes. GO (Gene Ontology) annotations were performed with InterProScan. GO enrichment for gene sets was conducted with Tbtools v1.098769 [87].

### Identification of the *AaTPS* gene in ISO-seq data

To identify *Aa*TPS proteins, the hidden Markov model (HMM) profiles of glycosyltransferase family 20 (Glyco_transf_20) (PF00982) and trehalose-phosphatase (Trehalose_PPase) (PF02358) were downloaded from InterPro (http://www.ebi.ac.uk/interpro/) and used to search using HMMER 3.0 [88]. The protein sequences of all *Aa*TPS family members were aligned using Muscle [89] with default parameters in MEGA7.0 [90]. Due to the structural similarity between TPP and TPS genes [15], we downloaded amino acid sequences for 11 *At*TPS and 10 *At*TPP [13] proteins from the National Center for Biotechnology Information (NCBI) and pooled them with sequences of 49 *Aa*TPS and *Aa*TPP proteins. A phylogenetic tree was constructed using neighbor-joining (NJ) method [91]. The bootstrap values for the phylogenetic trees were based on 1000 replicates, and pairwise deletion and the Poisson model were introduced.

### Auxin concentration measurement

Leaf buds and flower buds in Stage I were collected for IAA concentration detection. Two grams of sample were ground using a pre-cooled mortar and liquid nitrogen, and transferred into a tube containing 5 mL cold 80% (v/v) methanol for 4 h at 4 ℃. The mixture was then centrifuged for 15 min at 5000 rpm, and 4 ℃, and then the supernatant was collected. The supernatant is transported through a C18 Sep-Pak column (Waters, USA) and then dried with a vacuum evaporation device at 40 ℃. One milliliter of 60% methanol was used as the mobile phase to dissolve the extract. The solution was filtered through a 0.25 μm millipore filter for high performance liquid chromatography (HPLC) analysis. The parameters were set as follows: solution volume, 20 µL; chromatographic column model, ZORBAX SB-C18 (4.6 × 250 mm, 5 μm); mobile phase, 60% (v/v) methanol; flow rate, 0.5 mL/min; column temperature, 45 ℃; measure wavelength, 270 nm.

### Yeast two-hybrid screening

For the yeast two-hybrid screening, *AaSPL12* cDNA was introduced into pGBKT7 vector to generate the bait construct BD-*AaSPL12*. BD-*Aa*SPL12 plasmid was then transformed into the yeast strain AH109. The Anthurium cDNA library which was constructed into pGADT7 vector was transformed into the yeast cells that possesses the *BD*-*AaSPL12* plasmid. The transformants were selected on SD-TLH (-trp, -leu, -his) agar medium for 3–5 days. A total of 96 randomly picked positive clones were isolated and confirmed by PCR testing. These clones were then amplified using the Yeast Colony Rapid Detection Kit (Nanjing Ruiyuan Biotechnology Co., Ltd., Nanjing, China). The obtained sequences were aligned by Seqman and BLAST. The screened clones were diluted with sterile water and further cultivated on SD-TL (-trp, -leu), SD-TLH, SD-TLHA (-trp, -leu, -his, -ade) and SD-TLHA + X-α-gal for 3–4 days. All the positive clones were scraped using liquid 2×YPDA. Next Generation Sequencing (NGS) was taken for colony PCR production. The full-length of *AaSPL12* cDNA was amplified using the primers described in Table S1.

### Statistical analysis

The experimental data assessment on physiological and biochemical results was performed using a one-way ANOVA, followed by LSD *post hoc* analysis using Statistical Analysis System 9.4 (SAS). *P* < 0.05 was considered significant.

### Electronic supplementary material

Below is the link to the electronic supplementary material.

Supplementary Material 1

## Data Availability

The raw data of both RNA-seq and ISO-seq reported in this paper have been deposited in the China National GeneBank DataBase under project number CNP0004796. They are publicly accessible at https://db.cngb.org/.

## Declarations

### Supplementary information

**Additional file 1**

**Supplementary Figure S1.** Flower bud of Anthurium. **Supplementary Figure S2.** Isotope labelling of Anthurium leaf in different stages. **Supplementary Figure S3.** Phylogenetic tree of different TPS and TPP protein family members. **Supplementary Table S1.** Primer pairs used for detection of mRNAs. Primer of vectors used in yeast two-hybrid assay. **Supplementary 5.** Sequence information.
