# Supplementary material for: Auxin and carbohydrate control flower bud development in Anthurium andraeanum during early stage of sexual reproduction
Source: BMC Plant Biol. 2024 Mar 2;24:159. doi: 10.1186/s12870-024-04869-0 (PMC10908059; doi:10.1186/s12870-024-04869-0)
Supplement: Supplementary file 1 — Supplementary Material 1 [file 12870_2024_4869_MOESM1_ESM.docx]

**Supplementary Figure S1.**


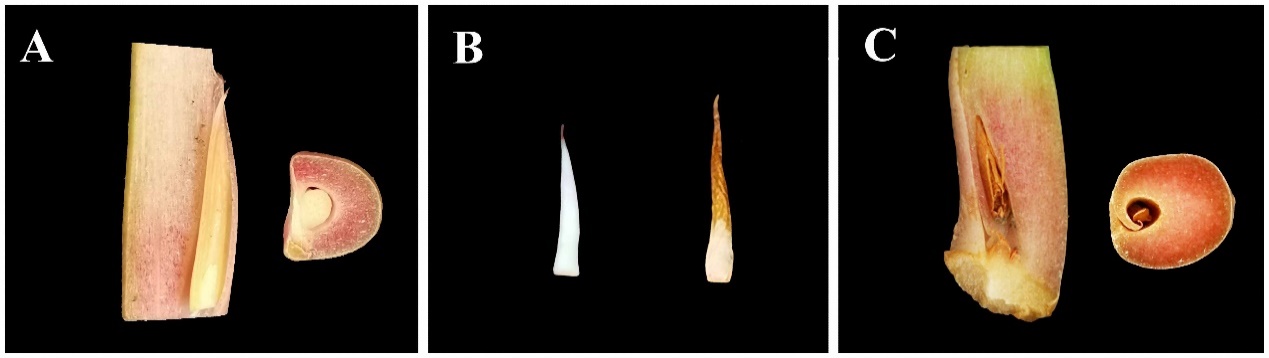


**Figure S1.** **Flower bud of *Anthurium andraeanum*.** (A) A normal flower bud. On the left side is a vertical section of flower bud stipule; while the right side is a cross-section of flower bud stipule. (B) Flower buds’ abortion which is in progress. The left bud shows the onset of abortion, and the right one exhibits a pronounced cell apoptosis. (C) Entirely abortive flower bud. The left side displays a vertical section of the stipule with an abortive flower bud; the right one shows a cross section of the stipule with an abortive flower bud.

**Supplementary Figure S2.**


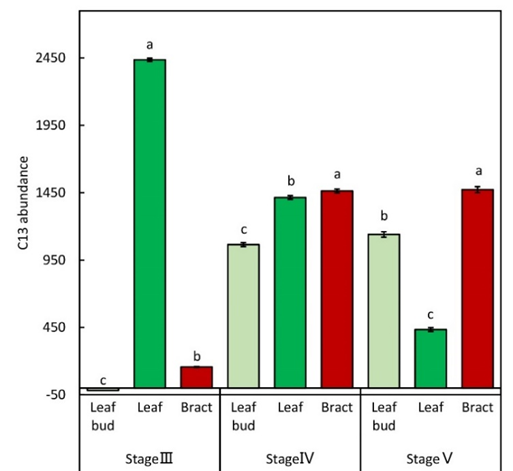


**Figure S2. Isotope labelling of *Anthurium* leaf in different stages.** Data are presented as means ± SEM. The different parts effect was evaluated by ANOVA one-way followed by LSD post hoc analysis. *P*＜0.05 was considered significant.

**Supplementary Figure S3.**


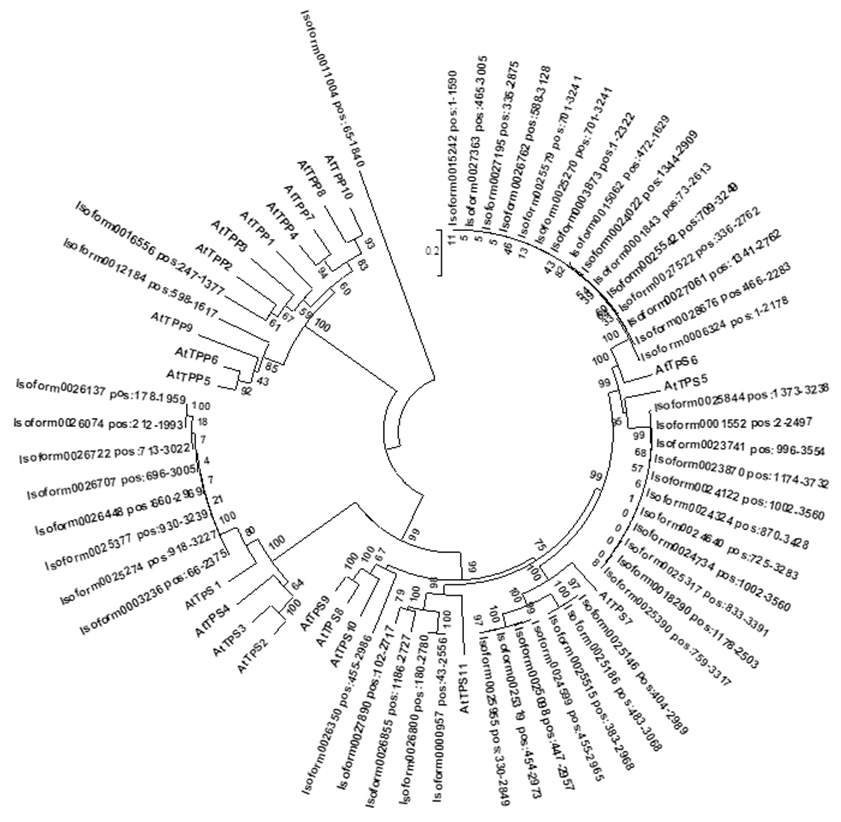


**Figure S3. Phylogenetic tree of different TPS and TPP protein family members**. Full-length sequences of 70 proteins from *Anthurium andraeanum* and Arabidopsis were used to construct the phylogenetic tree using MEGA7 with the NJ method.

**Supplementary Table S1**. Primers used for detection of mRNAs and primers used for vector constructions in Y2H assays.

| **Genes** | **Reference NO.** | **Primer 1** | **Primer 2** |
| --- | --- | --- | --- |
| Primers used for detection of mRNAs. | | | |
| AaTPS1 | Isoform0003236 | CGGTCACCACACGAAAACAT | CGACTCTGCTAGCTCCTTGA |
| AaARF2 | Isoform0028818 | ATATCTCTGCCGCGAAAGGA | TTTCCGGCACAAGAGAAAGC |
| AaARF2 | Isoform0002057 | ACAGAGTGGAGATTTCGGCA | TGTTATGGGACTCGGGCTTT |
| AaARF5 | Isoform0023604 | ACTTGTACAGCAGCAACAGC | ACTGGATGGAGGTTCAGGTG |
| AaARP | Isoform0023130 | CTTGCCTCCTCTCCCAAAGA | GCTCCTCCACACGTTATCCT |
| AaSPL12 | Isoform0025211 | GACGGTGATCTCTTTGTCGC | GTCCAAAACAACCACCTCCC |
| UBQ |  | TCCGCATCCAGAAGTGGTAC | TGCCGTCGTGGATCTCGTAG |
| Primers used for vector constructions in Y2H assays | | | |
| AaARP | Isoform0023130 | agaggaggacctgcatatgATGGAGGCCGAAATCGGCGCTG | cggcctccatggccatatgTTACATGAAACCGTAGTCCAAC |
| AaSPL12 | Isoform0025211 | accagattacgctcatatgAAGGAAGATGGTGCTGCTAGAC | tggcctccatggccatatgTCAGAGAGAGTGAGAGAGAGGA |

**Supplementary 5**. Sequence information.

>Isoform0002057 pos:306-1817

ATGGCGGGGATCGACCTCAACATGGTGGAGGACGGCGACGGCGCCGCGGGGGAGACCCAC

GGAGCGCAGCGGCAGGCGCACCACCCACCGCTTTCCTCCCCCTCCTCCGTCTGCTTGGAG

CTGTGGCACGCATGCGCGGGGCCGCTGATATATCTGCCACGCAGGGGGACCGTGGTGGTC

TACTTGCCGCAGGGTCATTTGGAGCAGATGGCGGGGGACTGCGCCGGCGCTGCCGCCGGG

GGTGGGGGAGCTGTGGGGGCGTCTTCCAGTTCCCGATACGACGTGCCTCCGCACGTCTTC

TGCCGGGTCATCGACGTCAAGCTCCATGCGGAGGCGGCCACGGACGAGGTGTACGCGCAG

CTTTCTCTTGCCCCCGAAAGTGAGGAGTTTGAAAAGCAGCTGCAAGAAGGTGAGGTTGAG

GAAGAAGGGGACACAGAGGAAGTTAGTGCCGTAACCAAGTCACTTACCCGTCATATGTTC

TGCAAGACGCTCACTGCCTCTGATACAAGCACACATGGAGGCTTCTCTGTCCCACGTAGA

GCTGCTGAAGATTGTTTCCCTCCACTGGATTATAAGCAACAGAGGCCCTCACAAGAGCTT

ATAGCAAAAGATTTGCATGGGACAGAGTGGAGATTTCGGCATATCTATAGGGGTCAGCCA

CGTAGGCACTTGCTCACAACAGGGTGGAGTGCATTTGTCAATAAGAAGAAGCTTATCTCA

GGGGATGCTGTGCTTTTTCTCAGAGGTGATGACGGAGAACTGAGATTAGGAATCCGGAGG

GCAGCTCCACTTAAGAGCAACGTTCCATATTCTACTCTTTCTGGTCAAAGCCCGAGTCCC

ATAACATTTTCTGCTGTGGTCAATTCCGTGTCATCAAAAAGTGTATTCCATGTTTACTAT

AATCCAAGGACCAGGCCATCAGATTTCATAGTACCTTATGGGAGGTTCACAAGGAGCCTG

AATAATTCATTTGCAATTGGAATGAGATTCAAGATGCATTGTGAAGGTGAAGATGCTGCA

GAGCGCAGGTACACGGGATTGATAACTGGAGTTTGTGATAAAGACCCTGTCAGATGGCCT

GGTTCAAAGTGGAAATGCCTTCTGGTGAGGTGGGACGATGACATTGAGGCAAACCGGCAG

AACAGGGTGTCTCCATGGGAAATAGAGCCATCTGGTTCAATCGGAAATTCCAATTGCCCA

CCAGCACCTGGTTCAAAGAGAACTAAGATCAACCTTAACTCACTCAATCCGAATTTCCCA

GTTCCTAGTGAAAGTGGGTGTCCAGACTTTGGGGAATCTGCTAGGTTCCATAAGGTCTTG

CAAGGTCAAGAAGTTTTGGGTTTTAGATCTCCTTATGATGGACTCAGTGCACCAAATCCT

CAGTTTTCCGAAATAAGGAGATGCATTCCTGATGCTAGCTGCCGTCTGTTAGCTACAGCC

AGGAACAATGTCAGAGTTCCTTTGGGAAAATCTGACATTTCCTATAAAGGCATAGGCTTT

GGAGAATCTGCT

>Isoform0001913 pos:117-1628

ATGGCGGGGATCGACCTCAACATGGTGGAGGACGGCGACGGCGCCGCGGGGGAGACCCAC

GGAGCGCAGCGGCAGGCGCACCACCCACCGCTTTCCTCCCCCTCCTCCGTCTGCTTGGAG

CTGTGGCACGCATGCGCGGGGCCGCTGATATATCTGCCACGCAGGGGGACCGTGGTGGTC

TACTTGCCGCAGGGTCATTTGGAGCAGATGGCGGGGGACTGCGCCGGCGCTGCCGCCGGG

GGTGGGGGAGCTGTGGGGGCGTCTTCCAGTTCCCGATACGACGTGCCTCCGCACGTCTTC

TGCCGGGTCATCGACGTCAAGCTCCATGCGGAGGCGGCCACGGACGAGGTGTACGCGCAG

CTTTCTCTTGCCCCCGAAAGTGAGGAGTTTGAAAAGCAGCTGCAAGAAGGTGAGGTTGAG

GAAGAAGGGGACACAGAGGAAGTTAGTGCCGTAACCAAGTCACTTACCCGTCATATGTTC

TGCAAGACGCTCACTGCCTCTGATACAAGCACACATGGAGGCTTCTCTGTCCCACGTAGA

GCTGCTGAAGATTGTTTCCCTCCACTGGATTATAAGCAACAGAGGCCCTCACAAGAGCTT

ATAGCAAAAGATTTGCATGGGACAGAGTGGAGATTTCGGCATATCTATAGGGGTCAGCCA

CGTAGGCACTTGCTCACAACAGGGTGGAGTGCATTTGTCAATAAGAAGAAGCTTATCTCA

GGGGATGCTGTGCTTTTTCTCAGAGGTGATGACGGAGAACTGAGATTAGGAATCCGGAGG

GCAGCTCCACTTAAGAGCAACGTTCCATATTCTACTCTTTCTGGTCAAAGCCCGAGTCCC

ATAACATTTTCTGCTGTGGTCAATTCCGTGTCATCAAAAAGTGTATTCCATGTTTACTAT

AATCCAAGGACCAGGCCATCAGATTTCATAGTACCTTATGGGAGGTTCACAAGGAGCCTG

AATAATTCATTTGCAATTGGAATGAGATTCAAGATGCATTGTGAAGGTGAAGATGCTGCA

GAGCGCAGGTACACGGGATTGATAACTGGAGTTTGTGATAAAGACCCTGTCAGATGGCCT

GGTTCAAAGTGGAAATGCCTTCTGGTGAGGTGGGACGATGACATTGAGGCAAACCGGCAG

AACAGGGTGTCTCCATGGGAAATAGAGCCATCTGGTTCAATCGGAAATTCCAATTGCCCA

CCAGCACCTGGTTCAAAGAGAACTAAGATCAACCTTAACTCACTCAATCCGAATTTCCCA

GTTCCTAGTGAAAGTGGGTGTCCAGACTTTGGGGAATCTGCTAGGTTCCATAAGGTCTTG

CAAGGTCAAGAAGTTTTGGGTTTTAGATCTCCTTATGATGGACTCAGTGCACCAAATCCT

CAGTTTTCCGAAATAAGGAGATGCATTCCTGATGCTAGCTGCCGTCTGTTAGCTACAGCC

AGGAACAATGTCAGAGTTCCTTTGGGAAAATCTGACATTTCCTATAAAGGCATAGGCTTT

GGAGAATCTGCT

>Isoform0001348 pos:240-2438

ATGACGGGGATCGACCTCAACACGGTGGAAGAGGACGACGGCGCCGGGGAGGGCCCGGTG

GCGACCCACCACCCGCCACCCTCCACGCCCACCGCCTCCTCTGTTTGCCTGGAGCTGTGG

CACGCCTGCGCAGGCCCGCTGATATCTCTGCCGCGAAAGGAGAGCGTGGTGGTCTACCTG

CCCCAGGGGCATCTGGAGCAGATGGTGGGTGAGTGCGGCGGGAATGGAGGAGCTGGCGGT

GGACCGTTCCGATGCAGTATGCCTCCACACGTCTTCTGCCGCGTCGTCGACGTCAAGCTC

CATGCAGAGGCCACCACGGACGACGTGTATGCACAGCTTTCTCTTGTGCCGGAAAGCGAG

GGATTCGAGCAGCAACTGCAAGAAGGAAGAGTGGAGGAAGAGGGTGACACTGAGGAACTC

TGTGGTGTAACTAAGTCATCAGTTATGCCTCATATGTTCTGCAAGACACTCACTGCCTCC

GATACGAGCACACATGGAGGCTTCTCTGTCCCACGTAGAGCTGCAGAGGACTGTTTTCCC

CCACTGGATTATAAACAACAGAGGCCTTCACAAGAACTTGCAGCAAAAGATTTGCATGGG

ACAGAGTGGAGGTTTCGGCACATCTACAGGGGTCAACCGCGTAGGCACTTGCTCACAACA

GGTTGGAGTGCATTTGTTAATAAGAAGAAACTTGTCTCTGGGGATGCTGTGCTCTTTCTT

AGAGGCGATGATGGGGTGTTGAGATTGGGACTCCGGAGGGCAGCTGCTCAATTTAAAAGT

AGTATTCCATGTTCGGTTCTTTGTGGTCAGAGTCTTAATCAGGGAACCTTTTTTGATGTG

GCGGATGCTGTGTCGTCAAGAAGTATGTTTCATATTTATTACAATCCGAGGGCAAGCACA

TCAGATTTCATAATTCCTTACTGGAAGTTCTCGAAGTGCATGAATAGTTCATTTTTAGTT

GGAATGAGGTTTAAGATGAGCTTTGAAAGTGAAGATGCTGCAGAACGGAGGTACACTGGA

GTGATAACTTGCATTGGTGATATGGACCCTGTCAGATGGCCTGGTTCAAAATGGAAATGC

TTTCTTGTGAGGTGGGATGACAATATTGAGACCAATCGGCAGAGTAGAGTTTCTCCATGG

GAAATAGAGCCATGCAATACAGTTGCAAGTTCCAGTTGTCTTGCAACTTCTGGTTCAAAG

AGAACCAAGATCAGTCTTACCTCTGTTGAATCAGATTTCACGTTTCCTAATGGAACTGGG

TTTCCAGTCTTTGGGGAATCTGCAAGATTCCGTAAGGTCTTGCAAGGTCAAGAAGTTTTG

GGTTTTAGACCTCCTTGTGGTGGTCTCGAGGCACTAAGTCGTCGGTCTTCTGAGAGGAGA

TGTACTGCTGCTAGCTGCCATTTGTTGGCTGCAGCAAAGAAAAATGTGGGATTTTCCGAA

GTGGAATCCGACATATCCTGCAAAGGTGCTGGCTTTCGAGAATCTGTTAGATTCCCTAAG

GTCTTGCAAGGTCAAGAAAAATTTCCTTTCAAACCCCCATATGAGGGGGGACAAGGTGAA

GGCCAGGCTGACAAAAATGCTGGGAACAGATGGCCTCGATTATATGGTTATAGTTCTTTG

GTGCGACAACAGGCAACCCCTTCCATGAAGGTTTCTTCGCCATCTTCTGTGCTAATGTTT

CAGCATGCAATTTCTCAGCTTCCATGCAATCGATCATTCCATAGGGCCGATGATTTGCAT

GCTGTTGTGGATGCCAACTATAGTGGTACTTTTGACTGCTTTGAAGCCTCAAGTGGGGAA

CACACATCATTGTTTTATCCACGTCAATGTATGGGTAACAATGTGGGAGCAGTATATTCA

TCACATTATCTTGGAATTAATAAATTGGCTGCAAATCATGCTCCTATACTGGTTTCTAAA

GCACATTCAGTAGAGGGCCAGGATTCTTCTTGCCACAGTGGAAGCAGTTGCAGACTTTTT

GGTTTTTCATTGACAGACACAAATCAAGAAAGAAATGGAGCGTTTGGCACACCATCTCCT

CTTCAGTCTTTAACTGATGCTAATACAGAGTCTTCCTCTCCTCCATCTGTGTCTCAAACA

TTGACCATGCCTAGGGAACACAGATGCAATGTAGTAGACACACTCCATCATGTGTGCGGT

GCACCAATTGGTAGATCTCCTGACCATTTCACTTTGTTA

>Isoform0028818 pos:414-2609

ATGACGGGGATCGACCTCAACACGGTGGAAGAGGACGACGGCGCCGGGGAGGGCCCGGTG

GCGACCCACCACCCGCCACCCTCCACGCCCACCGCCTCCTCTGTCTGCCTGGAGCTGTGG

CACGCCTGCGCAGGCCCGCTGATATCTCTGCCGCGAAAGGAGAGCGTGGTGGTCTACCTG

CCCCAGGGGCATCTGGAGCAGATGGTTGGTGAGTGCGGCGGGAATGGAGGAGCTGGCGGT

GGACCGTTCCGATGCAGTATGCCTCCACACGTCTTCTGCCGCGTCGTCGACGTCAAGCTC

CATGCAGAGGCCACCACGGACGACGTGTATGCACAGCTTTCTCTTGTGCCGGAAAGCGAG

GGATTCGAGCAGCAACTGCAAGAAGGAAGAGTGGAGGAAGAGGGTGACACTGAGGAACTC

TGTGGTGTAACTAAGTCATCAGTTATGCCTCATATGTTCTGCAAGACACTCACTGCCTCC

GATACGAGCACACATGGAGGCTTCTCTGTCCCACGTAGAGCTGCAGAGGACTGTTTTCCC

CCACTGGATTATAAACAACAGAGGCCTTCACAAGAACTTGCAGCAAAAGATTTGCATGGG

ACAGAGTGGAGGTTTCGGCACATCTACAGGGGTCAACCGCGTAGGCACTTGCTCACAACA

GGTTGGAGCGCATTTGTTAATAAGAAGAAACTTGTCTCTGGGGATGCTGTGCTCTTTCTT

AGAGGCGATGATGGGGTGTTGAGATTGGGACTCCGGAGGGCAGCTGCTCAATTTAAAAGT

AGTATTCCATGTTCGGTTCTTTGTGGTCAGAGTCTTAATCAGGGAACCTTTTTTGATGTG

GCGGATGCTGTGTCGTCAAGAAGTATGTTTCATATTTATTACAATCCGAGGGCAAGCACA

TCAGATTTCATAATTCCTTACTGGAAGTTCTCGAAGTGCATGAATAGTTCATTTTCAGTT

GGAATGAGGTTTAAGATGAGCTTTGAAAGTGAAGATGCTGCAGAACGGAGGTACACTGGA

GTGATAACTGGCATTGGTGATATGGACCCTATCAGATGGCCTGGTTCAAAATGGAAATGC

TTTCTTGTGAGGTGGGATGACAATATTGAGACCAATCGGCAGAGTAGAGTTTCTCCATGG

GAAATAGAGCCATGCAATACAGTTGCAAGTTCCAGTTGTCTTGCAACTTCTGGTTCAAAG

AGAACCAAGATCAGTCTTACCTCTGTTGAATCAGATTTCACGTTTCCTAATGGAACTGGG

TTTCCAGTCTTTGGGGAATCTGCAAGATTCCATAAGGTCTTGCAAGGTCAAGAAGTTTTG

GGTTTTAGACCTCCTTGTGGTGGTCTCGAGGCACTAAGTCGTCGGTCTTCTGAGAGGAGA

TGTACTGCTGCTAGCTGCCATTTGTTGGCTGCAGCAAAGAAAAATGTTGGATTTTCTGAA

GTGGAATCCGACATATCCTGCAAAGGTGCAGGCTTTCGAGAATCTGTTAGATTCCCTAAG

GTCTTGCAAGGTCAAGAAAAATTTCCTTTCAAACCCCCATATGAGGGGGGACAAGGTGAA

GGCCAGGCTGACAAAAATGCTGGGAACAGATGGCCTCGATTATATGGTTATAGTTCTTTG

GTGCGACAACAGGCAACCCCTTCCATGAAGGTTTCTTCGCCATCTTCTGTGCTAATGTTT

CAGCATGCAATTTCTCAGCTTCCATGCAATCGATCATTCCATAGGGCCGATGATTTGCAT

GCTGTTGTGGATGCCAACTATAGTGGTACTTTTGACTGCTTTGAAGCCTCAAGTGGGGAA

CACACATCATTGTTTTATCCACGTCAATGTATGGGTAACAATGTGGGAGCAGTATATTCA

TCACATTATCTTGGAATTAATAAATTGGCTGCAAATCATGCTCCTATACTGGTTTCTAAA

GCACATTCAGTAGAGGGCCAGGATTCTTCTTGCCACAGTGGAAGCAGTTGCAGACTTTTT

GGTTTTTCATTGACAGACACAAATCAAGAAAGAAATGGAGCGTTTAGCACACCATCTCTT

CAGTCTTTAACTGATGCTAATACAGAGTCTTCCTCTCCTCCATCTGTGTCTCAAACATTG

ACCATGCCTAGGGAACACAGATGCAATGTAGTAGACACACTCCATCATGTGTGCGGTGCA

CCAATTGGTAGATCTCCTGACCATTTCACTTTGTTA

>Isoform0004389 pos:10-2208

ATGACGGGGATCGACCTCAACACGGTGGAAGAGGACGACGGCGCCGGGGAGGGCCCGGTG

GCGACCCACCACCCGCCACCCTCCACGCCCACCGCCTCCTCTGTTTGCCTGGAGCTGTGG

CACGCCTGCGCAGGCCCGCTGATATCTCTGCCGCGAAAGGAGAGCGTGGTGGTCTACCTG

CCCCAGGGGCATCTGGAGCAGATGGTGGGTGAGTGCGGCGGGAATGGAGGAGCTGGCGGT

GGACCGTTCCGATGCAGTATGCCTCCACACGTCTTCTGCCGCGTCGTCGACGTCAAGCTC

CATGCAGAGGCCACCACGGACGACGTGTATGCACAGCTTTCTCTTGTGCCGGAAAGCGAG

GGATTCGAGCAGCAACTGCAAGAAGGAAGAGTGGAGGAAGAGGGTGACACTGAGGAACTC

TGTGGTGTAACTAAGTCATCAGTTATGCCTCATATGTTCTGCAAGACACTCACTGCCTCC

GATACGAGCACACATGGAGGCTTCTCTGTCCCACGTAGAGCTGCAGAGGACTGTTTTCCC

CCACTGGATTATAAACAACAGAGGCCTTCACAAGAACTTGCAGCAAAAGATTTGCATGGG

ACAGAGTGGAGGTTTCGGCACATCTACAGGGGTCAACCGCGTAGGCACTTGCTCACAACA

GGTTGGAGTGCATTTGTTAATAAGAAGAAACTTGTCTCTGGGGATGCTGTGCTCTTTCTT

AGAGGCGATGATGGGGTGTTGAGATTGGGACTCCGGAGGGCAGCTGCTCAATTTAAAAGT

AGTATTCCATGTTCGGTTCTTTGTGGTCAGAGTCTTAATCAGGGAACCTTTTTTGATGTG

GCGGATGCTGTGTCGTCAAGAAGTATGTTTCATATTTATTACAATCCGAGGGCAAGCACA

TCAGATTTCATAATTCCTTACTGGAAGTTCTCGAAGTGCATGAATAGTTCATTTTTAGTT

GGAATGAGGTTTAAGATGAGCTTTGAAAGTGAAGATGCTGCAGAACGGAGGTACACTGGA

GTGATAACTTGCATTGGTGATATGGACCCTGTCAGATGGCCTGGTTCAAAATGGAAATGC

TTTCTTGTGAGGTGGGATGACAATATTGAGACCAATCGGCAGAGTAGAGTTTCTCCATGG

GAAATAGAGCCATGCAATACAGTTGCAAGTTCCAGTTGTCTTGCAACTTCTGGTTCAAAG

AGAACCAAGATCAGTCTTACCTCTGTTGAATCAGATTTCACGTTTCCTAATGGAACTGGG

TTTCCAGTCTTTGGGGAATCTGCAAGATTCCGTAAGGTCTTGCAAGGTCAAGAAGTTTTG

GGTTTTAGACCTCCTTGTGGTGGTCTCGAGGCACTAAGTCGTCGGTCTTCTGAGAGGAGA

TGTACTGCTGCTAGCTGCCATTTGTTGGCTGCAGCAAAGAAAAATGTGGGATTTTCCGAA

GTGGAATCCGACATATCCTGCAAAGGTGCTGGCTTTCGAGAATCTGTTAGATTCCCTAAG

GTCTTGCAAGGTCAAGAAAAATTTCCTTTCAAACCCCCATATGAGGGGGGACAAGGTGAA

GGCCAGGCTGACAAAAATGCTGGGAACAGATGGCCTCGATTATATGGTTATAGTTCTTTG

GTGCGACAACAGGCAACCCCTTCCATGAAGGTTTCTTCGCCATCTTCTGTGCTAATGTTT

CAGCATGCAATTTCTCAGCTTCCATGCAATCGATCATTCCATAGGGCCGATGATTTGCAT

GCTGTTGTGGATGCCAACTATAGTGGTACTTTTGACTGCTTTGAAGCCTCAAGTGGGGAA

CACACATCATTGTTTTATCCACGTCAATGTATGGGTAACAATGTGGGAGCAGTATATTCA

TCACATTATCTTGGAATTAATAAATTGGCTGCAAATCATGCTCCTATACTGGTTTCTAAA

GCACATTCAGTAGAGGGCCAGGATTCTTCTTGCCACAGTGGAAGCAGTTGCAGACTTTTT

GGTTTTTCATTGACAGACACAAATCAAGAAAGAAATGGAGCGTTTGGCACACCATCTCCT

CTTCAGTCTTTAACTGATGCTAATACAGAGTCTTCCTCTCCTCCATCTGTGTCTCAAACA

TTGACCATGCCTAGGGAACACAGATGCAATGTAGTAGACACACTCCATCATGTGTGCGGT

GCACCAATTGGTAGATCTCCTGACCATTTCACTTTGTTA

>Isoform0027604 pos:561-2756

ATGACGGGGATCGACCTCAACACGGTGGAAGAGGACGACGGCGCCGGGGAGGGCCCGGTG

GCGACCCACCACCCGCCACCCTCCACGCCCACCGCCTCCTCTGTCTGCCTGGAGCTGTGG

CACGCCTGCGCAGGCCCGCTGATATCTCTGCCGCGAAAGGAGAGCGTGGTGGTCTACCTG

CCCCAGGGGCATCTGGAGCAGATGGTTGGTAAGTGCGGCGGGAATGGAGGAGCTGGCGGT

GGACCGTTCCGATGCAGTATGCCTCCACACGTCTTCTGCCGCGTCGTCGACGTCAAGCTC

CATGCAGAGGCCACCACGGACGACGTGTATGCACAGCTTTCTCTTGTGCCGGAAAGCGAG

GGATTCGAGCAGCAACTGCAAGAAGGAAGAGTGGAGGAAGAGGGTGACACTGAGGAACTC

TGTGGTGTAACTAAGTCATCAGTTATGCCTCATATGTTCTGCAAGACACTCACTGCCTCC

GATACGAGCACACATGGAGGCTTCTCTGTCCCACGTAGAGCTGCAGAGGACTGTTTCCCC

CCACTGGATTATAAACAACAGAGGCCTTCACAAGAACTTGCAGCAAAAGATTTGCATGGG

ACAGAGTGGAGGTTTCGGCACATCTACAGGGGTCAACCGCGTAGGCACTTGCTCACAACA

GGTTGGAGCGCATTTGTTAATAAGAAGAAACTTGTCTCTGGGGATGCTGTGCTCTTTCTT

AGAGGCGATGATGGGGTGTTGAGATTGGGACTCCGGAGGGCAGCTGCTCAATTTAAAAGT

AGTATTCCATGTTCGGTTCTTTGTGGTCAGAGTCTTAATCAGGGAACCTTTTTTGATGTG

GCGGATGCTGTGTCGTCAAGAAGTATGTTTCATATTTATTACAATCCGAGGGCAAGCACA

TCAGATTTCATAATTCCTTACTGGAAGTTCTCGAAGTGCATGAATAGTTCATTTTCAGTT

GGAATGAGGTTTAAGATGAGCTTTGAAAGTGAAGATGCTGCAGAACGGAGGTACACTGGA

GTGATAACTGGCATTGGTGATATGGACCCTATCAGATGGCCTGGTTCAAAATGGAAATGC

TTTCTTGTGAGGTGGGATGACAATATTGAGACCAATCGGCAGAGTAGAGTTTCTCCATGG

GAAATAGAGCCATGCAATACAGTTGCAAGTTCCAGTTGTCTTGCAACTTCTGGTTCAAAG

AGAACCAAGATCAGTCTTACCTCTGTTGAATCAGATTTCACGTTTCCTAATGGAACTGGG

TTTCCAGTCTTTGGGGAATCTGCAAGATTCCATAAGGTCTTGCAAGGTCAAGAAGTTTTG

GGTTTTAGACCTCCTTGTGGTGGTCTCGAGGCACTAAGTCGTCGGTCTTCTGAGAGGAGA

TGTACTGCTGCTAGCTGCCATTTGTTGGCTGCAGCAAAGAAAAATGTTGGATTTTCTGAA

GTGGAATCCGACATATCCTGCAAAGGTGCAGGCTTTCGAGAATCTGTTAGATTCCCTAAG

GTCTTGCAAGGTCAAGAAAAATTTCCTTTCAAACCCCCATATGAGGGGGGACAAGGTGAA

GGCCAGGCTGACAAAAATGCTGGGAACAGATGGCCTCGATTATATGGTTATAGTTCTTTG

GTGCGACAACAGGCAACCCCTTCCATGAAGGTTTCTTCGCCATCTTCTGTGCTAATGTTT

CAGCATGCAATTTCTCAGCTTCCATGCAATCGATCATTCCATAGGGCCGATGATTTGCAT

GCTGTTGTGGATGCCAACTATAGTGGTACTTTTGACTGCTTTGAAGCCTCAAGTGGGGAA

CACACATCATTGTTTTATCCACGTCAATGTATGGGTAACAATGTGGGAGCAGTATATTCA

TCACATTATCTTGGAATTAATAAATTGGCTGCAAATCATGCTCCTATACTGGTTTCTAAA

GCACATTCAGTAGAGGGCCAGGATTCTTCTTCCCACAGTGGAAGCAGTTGCAGACTTTTT

GGTTTTTCATTGACAGACACAAATCAAGAAAGAAATGGAGCGTTTAGCACACCATCTCTT

CAGTCTTTAACTGATGCTAATACAGAGTCTTCCTCTCCTCCATCTGTGTCTCAAACATTG

ACCATGCCTAGGGAACACAGATGCAATGTAGTAGACACACTCCATCATGTGTGCGGTGCA

CCAATTGGTAGATCTCCTGACCATTTCACTTTGTTA

>Isoform0028390 pos:474-2672

ATGACGGGGATCGACCTCAACACGGTGGAAGAGGACGACGGCGCCGGGGAGGGCCCGGTG

GCGACCCACCACCCGCCACCCTCCACGCCCACCGCCTCCTCTGTTTGCCTGGAGCTGTGG

CACGCCTGCGCAGGCCCGCTGATATCTCTGCCGCGAAAGGAGAGCGTGGTGGTCTACCTG

CCCCAGGGGCATCTGGAGCAGATGGTGGGTGAGTGCGGCGGGAATGGAGGAGCTGGCGGT

GGACCGTTCCGATGCAGTATGCCTCCACACGTCTTCTGCCGCGTCGTCGACGTCAAGCTC

CATGCAGAGGCCACCACGGACGACGTGTATGCACAGCTTTCTCTTGTGCCGGAAAGCGAG

GGATTCGAGCAGCAACTGCAAGAAGGAAGAGTGGAGGAAGAGGGTGACACTGAGGAACTC

TGTGGTGTAACTAAGTCATCAGTTATGCCTCATATGTTCTGCAAGACACTCACTGCCTCC

GATACGAGCACACATGGAGGCTTCTCTGTCCCACGTAGAGCTGCAGAGGACTGTTTTCCC

CCACTGGATTATAAACAACAGAGGCCTTCACAAGAACTTGCAGCAAAAGATTTGCATGGG

ACAGAGTGGAGGTTTCGGCACATCTACAGGGGTCAACCGCGTAGGCACTTGCTCACAACA

GGTTGGAGTGCATTTGTTAATAAGAAGAAACTTGTCTCTGGGGATGCTGTGCTCTTTCTT

AGAGGCGATGATGGGGTGTTGAGATTGGGACTCCGGAGGGCAGCTGCTCAATTTAAAAGT

AGTATTCCATGTTCGGTTCTTTGTGGTCAGAGTCTTAATCAGGGAACCTTTTTTGATGTG

GCGGATGCTGTGTCGTCAAGAAGTATGTTTCATATTTATTACAATCCGAGGGCAAGCACA

TCAGATTTCATAATTCCTTACTGGAAGTTCTCGAAGTGCATGAATAGTTCATTTTTAGTT

GGAATGAGGTTTAAGATGAGCTTTGAAAGTGAAGATGCTGCAGAACGGAGGTACACTGGA

GTGATAACTTGCATTGGTGATATGGACCCTGTCAGATGGCCTGGTTCAAAATGGAAATGC

TTTCTTGTGAGGTGGGATGACAATATTGAGACCAATCGGCAGAGTAGAGTTTCTCCATGG

GAAATAGAGCCATGCAATACAGTTGCAAGTTCCAGTTGTCTTGCAACTTCTGGTTCAAAG

AGAACCAAGATCAGTCTTACCTCTGTTGAATCAGATTTCACGTTTCCTAATGGAACTGGG

TTTCCAGTCTTTGGGGAATCTGCAAGATTCCGTAAGGTCTTGCAAGGTCAAGAAGTTTTG

GGTTTTAGACCTCCTTGTGGTGGTCTCGAGGCACTAAGTCGTCGGTCTTCTGAGAGGAGA

TGTACTGCTGCTAGCTGCCATTTGTTGGCTGCAGCAAAGAAAAATGTGGGATTTTCCGAA

GTGGAATCCGACATATCCTGCAAAGGTGCTGGCTTTCGAGAATCTGTTAGATTCCCTAAG

GTCTTGCAAGGTCAAGAAAAATTTCCTTTCAAACCCCCATATGAGGGGGGACAAGGTGAA

GGCCAGGCTGACAAAAATGCTGGGAACAGATGGCCTCGATTATATGGTTATAGTTCTTTG

GTGCGACAACAGGCAACCCCTTCCATGAAGGTTTCTTCGCCATCTTCTGTGCTAATGTTT

CAGCATGCAATTTCTCAGCTTCCATGCAATCGATCATTCCATAGGGCCGATGATTTGCAT

GCTGTTGTGGATGCCAACTATAGTGGTACTTTTGACTGCTTTGAAGCCTCAAGTGGGGAA

CACACATCATTGTTTTATCCACGTCAATGTATGGGTAACAATGTGGGAGCAGTATATTCA

TCACATTATCTTGGAATTAATAAATTGGCTGCAAATCATGCTCCTATACTGGTTTCTAAA

GCACATTCAGTAGAGGGCCAGGATTCTTCTTGCCACAGTGGAAGCAGTTGCAGACTTTTT

GGTTTTTCATTGACAGACACAAATCAAGAAAGAAATGGAGCGTTTGGCACACCATCTCCT

CTTCAGTCTTTAACTGATGCTAATACAGAGTCTTCCTCTCCTCCATCTGTGTCTCAAACA

TTGACCATGCCTAGGGAACACAGATGCAATGTAGTAGACACACTCCATCATGTGTGCGGT

GCACCAATTGGTAGATCTCCTGACCATTTCACTTTGTTA

>Isoform0026137 pos:178-1959

ATGGCGGTGAACAAGTGCGGTGGTCGCGGCCCCAAGTCAAGGGTGGAGCGGCTGCTCATG

GAGAGAGAGCTGAGGAAGTTCAACAGGACCATCCACCTCAACGACTATGGCGGCGACTCT

GCCCGGGGTTTCGGCGGGTTGGAGCATGACCGAGGGAGCTGCGACAGCGGCGAGTGGGTG

ACTGTGGGTGAAGGTGAGATCTTTGATGCAGCCGCCGTCGCCTTGAGGGCGGCGAGCGAG

GGTTGGGACGACCAGGATGGGAAACTGCTGAAGCAGCGGTTGCTGGTGGTGGCAAACCGG

CTGCCGGTCTCTGCCGTCAGGAGAGCTGAGGATTCTTGGTCCCTGGAGATCAGTGCCGGT

GGACTTGTTAGTGCTCTTCTTGGTGTGAAGCAGTTCGAAGCTAGGTGGATAGGATGGGCT

GGTGTGAACGTACCAGATGAAATTGGGCAGAGAGCACTTACAAAGGCACTGGCTGAGAAG

ATGTGCATCCCTGTGTTTCTCGATGAAGAAATAGTTCATCAATATTACAATGGTTACTGC

AACAATGTATTGTGGCCATTATTTCACTATCTTGGGCTTCCGCAAGAGGATCGTTTGGCA

ACTACCCGTAGTTTCCAATCCCAGTTTGATGCTTACAAGCGTGCAAACCAAATGTTTGCT

GACGTGGTGAACCAACACTATGAAGAGGGAGATGTGGTTTGGTGCCATGACTACCATCTT

ATGTACCTCCCAAAATGCCTTAAAGAGTATAACAGGCAAATGAAAGTTGGATGGTTCCTT

CATACACCCTTTCCCTCCTCCGAAATTTACAGGACATTGCCATCACGATCAGAGTTGTTG

AGATCTGTTCTTGCTGCTGATTTGGTCGGATTTCACACTTACGATTATGCAAGGCATTTT

GTTAGTGCCTGTACGCGGATTCTGGGATTTGAAGGCACCCCTGAAGGGGTGGAGGATCAA

GGAAGGTTGACTCGAGTTGCTGCATTTCCTATTGGGATAGACTCTGACCGTTTCACTCAA

GCACTTGAGCTTCCAAAAGTTAAAGATCACATGAAAGATTTAAGAGAGAGATTTGCTGGC

CGAAAGGTGATGTTAGGTGTTGACCGACTCGACATGATTAAAGGAATACCTCAGAAAATT

TTGGCATTCGAAAAGTTCCTTGAGGAAAATCCTTCTTGGTGTGATAAAGTAGTTCTATTG

CAAATAGCTGTGCCAACTAGAACAGATGTTCCTGAATATCAAAAACTTACCAGCCAGGTC

CATGAAATTGTTGGACGCATTAATGGCCGTTTTGGAACACTCTCTGCAGTTCCTATACAT

CATCTGGATCGGTCTCTTGACTTCAATGCATTATGTGCACTCTATGCAGTCACTGATGTG

GCTCTTGTAACATCACTGAGGGATGGGATGAATCTAGTAAGCTATGAATTTGTGGCTTGC

CAAGAGCAAAAGAAGGGGGTTCTCATTCTCAGTGAATTTGCAGGAGCTGCACAATCTCTG

GGTGCTGGAGCACTACTTGTAAACCCATGGAACATCACTGAAGTTGCTTACTCGATAGGC

TATGCGTTGAATATGCCTCCGGATGAAAGAGAAAAGAGACACAGGCATAACTTTGCTCAT

GTCACCACCCACACTGCTCAAGATTGGGCTGAAACTTTTGTAAGTGAACTTAACGATACT

GTCTTTGAAGCTCAGCTTAGGACAAGACAGGTCCCACCTTTACTTCCAACAAGAATTGCT

ATGGAACGTTATTTTCAGTCCAAGAATCGATTGCTTATTCTGGGTTTCAATGCTACGTTGACT

GAACAAGTGGAGTCTCCTGGAAGGAGAGGTGGTGATCAGATTAAAGAGATGGAGCTTAAA

TTGCATCCAGAACTGAAACATCCCTTGAGCATACTTTGTAATGATCCAAAGACGACTATT

GTTGTCCTAAGTGGAAGTGACAGAAGTGTCCTGGATGATAACTTTGGAGAGTACAATATG

TGGTTGGCTGCAGAGAATGGGATGTTTTTACGTCTAACAACTGGAGAATGGATGACAACA

ATGCCAGAGCACCTAAACATGGATTGGGTTGAGAGCGTAAAGCATGTCTTTGAGTACTTC

ACCGAAAGAACACCTAGATCCCATTTTGAACACCGTGAGACTTCACTTTTGTGGAACTAT

AAGTATGCTGATGCTGAATTCGGAAGGCTTCAAGCAAGAGATATGTTGCAGCACTTGTGG

ACTGGACCGATTTCTAATGCTGCTGTAGATGTTGTCCAAGGCAGCCGATCTGTTGAGGTC

CGCTCTGTTGGTGTTACCAAGGGTGCAGCAATTGATCGTATCTTAGGGGAAATAGTTCAT

AGCCAATCTATGATTACACCAATCGATTATGTTTTATGCATCGGCCACTTTCTTGGAAAG

GATGAAGATATTTATACATTTTTTGAGCCAGAGCTTCCTGCTGAGCCTGTTGGCATTAGA

ACGAAGGCAGCTGAGATAATCAAGCCATCTATGGAGCGTAAAGTCTCCTCTAAGCTGTCA

AACATGAAGAACAACTCGAGGACAACTCAAGGTAAAGCACAGAAGGTCTCAACTGGAGCT

GACAGGAAAACTACTAGTAACTGCACTGTAACGGGATCTCGGTCACCACACGAAAACATC

TCATGTCATGAAGGATCATCTGTTCTGGACCTCAAAGGAGAGAATTACTTCTCGTGCGCA

GTAGGTCGGAAGTGTTCAAATGCCCGTTATCTGCTCGACACGTCTGATGATGTGGTCACA

TTTGTCAAGGAGCTAGCAGAGTCGTGTGGC

>Isoform0026074 pos:212-1993

ATGGCGGTGAACAAGTGCGGTGGTCGCGGCCCCAAGTCAAGGGTGGAGCGGCTGCTCATG

GAGAGAGAGCTGAGGAAGTTCAACAGGACCATCCACCTCAACGACTATGGCGGCGACTCT

GCCCGGGGTTTCGGCGGGTTGGAGCATGACCGAGGGAGCTGCGACAGCGGCGAGTGGGTG

ACTGTGGGTGAAGGTGAGATCTTTGATGCAGCCGCCGTCGCCTTGAGGGCGGCGAGCGAG

GGTTGGGACGACCAGGATGGGAAACTGCTGAAGCAGCGGTTGCTGGTGGTGGCAAACCGG

CTGCCGGTCTCTGCCGTCAGGAGAGCTGAGGATTCTTGGTCCCTGGAGATCAGTGCCGGT

GGACTTGTTAGTGCTCTTCTTGGTGTGAAGCAGTTCGAAGCTAGGTGGATAGGATGGGCT

GGTGTGAACGTACCAGATGAAATTGGGCAGAGAGCACTTACAAAGGCACTGGCTGAGAAG

ATGTGCATCCCTGTGTTTCTCGATGAAGAAATAGTTCATCAATATTACAATGGTTACTGC

AACAATGTATTGTGGCCATTATTTCACTATCTTGGGCTTCCGCAAGAGGATCGTTTGGCA

ACTACCCGTAGTTTCCAATCCCAGTTTGATGCTTACAAGCGTGCAAACCAAATGTTTGCT

GACGTGGTGAACCAACACTATGAAGAGGGAGATGTGGTTTGGTGCCATGACTACCATCTT

ATGTACCTCCCAAAATGCCTTAAAGAGTATAACAGGCAAATGAAAGTTGGATGGTTCCTT

CATACACCCTTTCCCTCCTCCGAAATTTACAGGACATTGCCATCACGATCAGAGTTGTTG

AGATCTGTTCTTGCTGCTGATTTGGTCGGATTTCACACTTACGATTATGCAAGGCATTTT

GTTAGTGCCTGTACGCGGATTCTGGGATTTGAAGGCACCCCTGAAGGGGTGGAGGATCAA

GGAAGGTTGACTCGAGTTGCTGCATTTCCTATTGGGATAGACTCTGACCGTTTCACTCAA

GCACTTGAGCTTCCAAAAGTTAAAGATCACATGAAAGATTTAAGAGAGAGATTTGCTGGC

CGAAAGGTGATGTTAGGTGTTGACCGACTCGACATGATTAAAGGAATACCTCAGAAAATT

TTGGCATTCGAAAAGTTCCTTGAGGAAAATCCTTCTTGGTGTGATAAAGTAGTTCTATTG

CAAATAGCTGTGCCAACTAGAACAGATGTTCCTGAATATCAAAAACTTACCAGCCAGGTC

CATGAAATTGTTGGACGCATTAATGGCCGTTTTGGAACACTCTCTGCAGTTCCTATACAT

CATCTGGATCGGTCTCTTGACTTCAATGCATTATGTGCACTCTATGCAGTCACTGATGTG

GCTCTTGTAACATCACTGAGGGATGGGATGAATCTAGTAAGCTATGAATTTGTGGCTTGC

CAAGAGCAAAAGAAGGGGGTTCTCATTCTCAGTGAATTTGCAGGAGCTGCACAATCTCTG

GGTGCTGGAGCACTACTTGTAAACCCATGGAACATCACTGAAGTTGCTTACTCGATAGGC

TATGCGTTGAATATGCCTCCGGATGAAAGAGAAAAGAGACACAGGCATAACTTTGCTCAT

GTCACCACCCACACTGCTCAAGATTGGGCTGAAACTTTTGTAAGTGAACTTAACGATACT

GTCTTTGAAGCTCAGCTTAGGACAAGACAGGTCCCACCTTTACTTCCAACAAGAATTGCT

ATGGAACGTTATTTTCAGTCCAAGAATCGATTGCTTATTCTGGGTTTCAATGCTACGTTGACT

GAACAAGTGGAGTCTCCTGGAAGGAGAGGTGGTGATCAGATTAAAGAGATGGAGCTTAAA

TTGCATCCAGAACTGAAACATCCCTTGAGCATACTTTGTAATGATCCAAAGACGACTATT

GTTGTCCTAAGTGGAAGTGACAGAAGTGTCCTGGATGATAACTTTGGAGAGTACAATATG

TGGTTGGCTGCAGAGAATGGGATGTTTTTACGTCTAACAACTGGAGAATGGATGACAACA

ATGCCAGAGCACCTAAACATGGATTGGGTTGAGAGCGTAAAGCATGTCTTTGAGTACTTC

ACCGAAAGAACACCTAGATCCCATTTTGAACACCGTGAGACTTCACTTTTGTGGAACTAT

AAGTATGCTGATGCTGAATTCGGAAGGCTTCAAGCAAGAGATATGTTGCAGCACTTGTGG

ACTGGACCGATTTCTAATGCTGCTGTAGATGTTGTCCAAGGCAGCCGATCTGTTGAGGTC

CGCTCTGTTGGTGTTACCAAGGGTGCAGCAATTGATCGTATCTTAGGGGAAATAGTTCAT

AGCCAATCTATGATTACACCAATCGATTATGTTTTATGCATCGGCCACTTTCTTGGAAAG

GATGAAGATATTTATACATTTTTTGAGCCAGAGCTTCCTGCTGAGCCTGTTGGCATTAGA

ACGAAGGCAGCTGAGATAATCAAGCCATCTATGGAGCGTAAAGTCTCCTCTAAGCTGTCA

AACATGAAGAACAACTCGAGGACAACTCAAGGTAAAGCACAGAAGGTCTCAACTGGAGCT

GACAGGAAAACTACTAGTAACTGCACTGTAACGGGATCTCGGTCACCACACGAAAACATC

TCATGTCATGAAGGATCATCTGTTCTGGACCTCAAAGGAGAGAATTACTTCTCGTGCGCA

GTAGGTCGGAAGTGTTCAAATGCCCGTTATCTGCTCGACACGTCTGATGATGTGGTCACA

TTTGTCAAGGAGCTAGCAGAGTCGTGTGGC

>Isoform0026722 pos:713-3022

TGCATCCCTGTGTTTCTCGATGAAGAAATAGTTCATCAATATTACAATGGTTACTGCAAC

AATGTATTGTGGCCATTATTTCACTATCTTGGGCTTCCGCAAGAGGATCGTTTGGCAACT

ACCCGTAGTTTCCAATCCCAGTTTGATGCTTACAAGCGTGCAAACCAAATGTTTGCTGAC

GTGGTGAACCAACACTATGAAGAGGGAGATGTGGTTTGGTGCCATGACTACCATCTTATG

TACCTCCCAAAATGCCTTAAAGAGTATAACAGGCAAATGAAAGTTGGATGGTTCCTTCAT

ACACCCTTTCCCTCCTCCGAAATTTACAGGACATTGCCATCACGATCAGAGTTGTTGAGA

TCTGTTCTTGCTGCTGATTTGGTCGGATTTCACACTTACGATTATGCAAGGCATTTTGTT

AGTGCCTGTACGCGGATTCTGGGATTTGAAGGCACCCCTGAAGGGGTGGAGGATCAAGGA

AGGTTGACTCGAGTTGCTGCATTTCCTATTGGGATAGACTCTGACCGTTTCACTCAAGCA

CTTGAGCTTCCAAAAGTTAAAGATCACATGAAAGATTTAAGAGAGAGATTTGCTGGCCGA

AAGGTGATGTTAGGTGTTGACCGACTCGACATGATTAAAGGAATACCTCAGAAAATTTTG

GCATTCGAAAAGTTCCTTGAGGAAAATCCTTCTTGGTGTGATAAAGTAGTTCTATTGCAA

ATAGCTGTGCCAACTAGAACAGATGTTCCTGAATATCAAAAACTTACCAGCCAGGTCCAT

GAAATTGTTGGACGCATTAATGGCCGTTTTGGAACACTCTCTGCAGTTCCTATACATCAT

CTGGATCGGTCTCTTGACTTCAATGCATTATGTGCACTCTATGCAGTCACTGATGTGGCT

CTTGTAACATCACTGAGGGATGGGATGAATCTAGTAAGCTATGAATTTGTGGCTTGCCAA

GAGCAAAAGAAGGGGGTTCTCATTCTCAGTGAATTTGCAGGAGCTGCACAATCTCTGGGT

GCTGGAGCACTACTTGTAAACCCATGGAACATCACTGAAGTTGCTTACTCGATAGGCTAT

GCGTTGAATATGCCTCCGGATGAAAGAGAAAAGAGACACAGGCATAACTTTGCTCATGTC

ACCACCCACACTGCTCAAGATTGGGCTGAAACTTTTGTAAGTGAACTTAACGATACTGTC

TTTGAAGCTCAGCTTAGGACAAGACAGGTCCCACCTTTACTTCCAACAAGAATTGCTATG

GAACGTTATTTTCAGTCCAAGAATCGATTGCTTATTCTGGGTTTCAATGCTACGTTGACT

GAACAAGTGGAGTCTCCTGGAAGGAGAGGTGGTGATCAGATTAAAGAGATGGAGCTTAAA

TTGCATCCAGAACTGAAACATCCCTTGAGCATACTTTGTAATGATCCAAAGACGACTATT

GTTGTCCTAAGTGGAAGTGACAGAAGTGTCCTGGATGATAACTTTGGAGAGTACAATATG

TGGTTGGCTGCAGAGAATGGGATGTTTTTACGTCTAACAACTGGAGAATGGATGACAACA

ATGCCAGAGCACCTAAACATGGATTGGGTTGAGAGCGTAAAGCATGTCTTTGAGTACTTC

ACCGAAAGAACACCTAGATCCCATTTTGAACACCGTGAGACTTCACTTTTGTGGAACTAT

AAGTATGCTGATGCTGAATTCGGAAGGCTTCAAGCAAGAGATATGTTGCAGCACTTGTGG

ACTGGACCGATTTCTAATGCTGCTGTAGATGTTGTCCAAGGCAGCCGATCTGTTGAGGTC

CGCTCTGTTGGTGTTACCAAGGGTGCAGCAATTGATCGTATCTTAGGGGAAATAGTTCAT

AGCCAATCTATGATTACACCAATCGATTATGTTTTATGCATCGGCCACTTTCTTGGAAAG

GATGAAGATATTTATACATTTTTTGAGCCAGAGCTTCCTGCTGAGCCTGTTGGCATTAGA

ACGAAGGCAGCTGAGATAATCAAGCCATCTATGGAGCGTAAAGTCTCCTCTAAGCTGTCA

AACATGAAGAACAACTCGAGGACAACTCAAGGTAAAGCACAGAAGGTCTCAACTGGAGCT

GACAGGAAAACTACTAGTAACTGCACTGTAACGGGATCTCGGTCACCACACGAAAACATC

TCATGTCATGAAGGATCATCTGTTCTGGACCTCAAAGGAGAGAATTACTTCTCGTGCGCA

GTAGGTCGGAAGTGTTCAAATGCCCGTTATCTGCTCGACACGTCTGATGATGTGGTCACA

TTTGTCAAGGAGCTAGCAGAGTCGTGTGGC

>Isoform0026707 pos:696-3005

TGCATCCCTGTGTTTCTCGATGAAGAAATAGTTCATCAATATTACAATGGTTACTGCAAC

AATGTATTGTGGCCATTATTTCACTATCTTGGGCTTCCGCAAGAGGATCGTTTGGCAACT

ACCCGTAGTTTCCAATCCCAGTTTGATGCTTACAAGCGTGCAAACCAAATGTTTGCTGAC

GTGGTGAACCAACACTATGAAGAGGGAGATGTGGTTTGGTGCCATGACTACCATCTTATG

TACCTCCCAAAATGCCTTAAAGAGTATAACAGGCAAATGAAAGTTGGATGGTTCCTTCAT

ACACCCTTTCCCTCCTCCGAAATTTACAGGACATTGCCATCACGATCAGAGTTGTTGAGA

TCTGTTCTTGCTGCTGATTTGGTCGGATTTCACACTTACGATTATGCAAGGCATTTTGTT

AGTGCCTGTACGCGGATTCTGGGATTTGAAGGCACCCCTGAAGGGGTGGAGGATCAAGGA

AGGTTGACTCGAGTTGCTGCATTTCCTATTGGGATAGACTCTGACCGTTTCACTCAAGCA

CTTGAGCTTCCAAAAGTTAAAGATCACATGAAAGATTTAAGAGAGAGATTTGCTGGCCGA

AAGGTGATGTTAGGTGTTGACCGACTCGACATGATTAAAGGAATACCTCAGAAAATTTTG

GCATTCGAAAAGTTCCTTGAGGAAAATCCTTCTTGGTGTGATAAAGTAGTTCTATTGCAA

ATAGCTGTGCCAACTAGAACAGATGTTCCTGAATATCAAAAACTTACCAGCCAGGTCCAT

GAAATTGTTGGACGCATTAATGGCCGTTTTGGAACACTCTCTGCAGTTCCTATACATCAT

CTGGATCGGTCTCTTGACTTCAATGCATTATGTGCACTCTATGCAGTCACTGATGTGGCT

CTTGTAACATCACTGAGGGATGGGATGAATCTAGTAAGCTATGAATTTGTGGCTTGCCAA

GAGCAAAAGAAGGGGGTTCTCATTCTCAGTGAATTTGCAGGAGCTGCACAATCTCTGGGT

GCTGGAGCACTACTTGTAAACCCATGGAACATCACTGAAGTTGCTTACTCGATAGGCTAT

GCGTTGAATATGCCTCCGGATGAAAGAGAAAAGAGACACAGGCATAACTTTGCTCATGTC

ACCACCCACACTGCTCAAGATTGGGCTGAAACTTTTGTAAGTGAACTTAACGATACTGTC

TTTGAAGCTCAGCTTAGGACAAGACAGGTCCCACCTTTACTTCCAACAAGAATTGCTATG

GAACGTTATTTTCAGTCCAAGAATCGATTGCTTATTCTGGGTTTCAATGCTACGTTGACT

GAACAAGTGGAGTCTCCTGGAAGGAGAGGTGGTGATCAGATTAAAGAGATGGAGCTTAAA

TTGCATCCAGAACTGAAACATCCCTTGAGCATACTTTGTAATGATCCAAAGACGACTATT

GTTGTCCTAAGTGGAAGTGACAGAAGTGTCCTGGATGATAACTTTGGAGAGTACAATATG

TGGTTGGCTGCAGAGAATGGGATGTTTTTACGTCTAACAACTGGAGAATGGATGACAACA

ATGCCAGAGCACCTAAACATGGATTGGGTTGAGAGCGTAAAGCATGTCTTTGAGTACTTC

ACCGAAAGAACACCTAGATCCCATTTTGAACACCGTGAGACTTCACTTTTGTGGAACTAT

AAGTATGCTGATGCTGAATTCGGAAGGCTTCAAGCAAGAGATATGTTGCAGCACTTGTGG

ACTGGACCGATTTCTAATGCTGCTGTAGATGTTGTCCAAGGCAGCCGATCTGTTGAGGTC

CGCTCTGTTGGTGTTACCAAGGGTGCAGCAATTGATCGTATCTTAGGGGAAATAGTTCAT

AGCCAATCTATGATTACACCAATCGATTATGTTTTATGCATCGGCCACTTTCTTGGAAAG

GATGAAGATATTTATACATTTTTTGAGCCAGAGCTTCCTGCTGAGCCTGTTGGCATTAGA

ACGAAGGCAGCTGAGATAATCAAGCCATCTATGGAGCGTAAAGTCTCCTCTAAGCTGTCA

AACATGAAGAACAACTCGAGGACAACTCAAGGTAAAGCACAGAAGGTCTCAACTGGAGCT

GACAGGAAAACTACTAGTAACTGCACTGTAACGGGATCTCGGTCACCACACGAAAACATC

TCATGTCATGAAGGATCATCTGTTCTGGACCTCAAAGGAGAGAATTACTTCTCGTGCGCA

GTAGGTCGGAAGTGTTCAAATGCCCGTTATCTGCTCGACACGTCTGATGATGTGGTCACA

TTTGTCAAGGAGCTAGCAGAGTCGTGTGGC

>Isoform0026448 pos:660-2969

TGCATCCCTGTGTTTCTCGATGAAGAAATAGTTCATCAATATTACAATGGTTACTGCAAC

AATGTATTGTGGCCATTATTTCACTATCTTGGGCTTCCGCAAGAGGATCGTTTGGCAACT

ACCCGTAGTTTCCAATCCCAGTTTGATGCTTACAAGCGTGCAAACCAAATGTTTGCTGAC

GTGGTGAACCAACACTATGAAGAGGGAGATGTGGTTTGGTGCCATGACTACCATCTTATG

TACCTCCCAAAATGCCTTAAAGAGTATAACAGGCAAATGAAAGTTGGATGGTTCCTTCAT

ACACCCTTTCCCTCCTCCGAAATTTACAGGACATTGCCATCACGATCAGAGTTGTTGAGA

TCTGTTCTTGCTGCTGATTTGGTCGGATTTCACACTTACGATTATGCAAGGCATTTTGTT

AGTGCCTGTACGCGGATTCTGGGATTTGAAGGCACCCCTGAAGGGGTGGAGGATCAAGGA

AGGTTGACTCGAGTTGCTGCATTTCCTATTGGGATAGACTCTGACCGTTTCACTCAAGCA

CTTGAGCTTCCAAAAGTTAAAGATCACATGAAAGATTTAAGAGAGAGATTTGCTGGCCGA

AAGGTGATGTTAGGTGTTGACCGACTCGACATGATTAAAGGAATACCTCAGAAAATTTTG

GCATTCGAAAAGTTCCTTGAGGAAAATCCTTCTTGGTGTGATAAAGTAGTTCTATTGCAA

ATAGCTGTGCCAACTAGAACAGATGTTCCTGAATATCAAAAACTTACCAGCCAGGTCCAT

GAAATTGTTGGACGCATTAATGGCCGTTTTGGAACACTCTCTGCAGTTCCTATACATCAT

CTGGATCGGTCTCTTGACTTCAATGCATTATGTGCACTCTATGCAGTCACTGATGTGGCT

CTTGTAACATCACTGAGGGATGGGATGAATCTAGTAAGCTATGAATTTGTGGCTTGCCAA

GAGCAAAAGAAGGGGGTTCTCATTCTCAGTGAATTTGCAGGAGCTGCACAATCTCTGGGT

GCTGGAGCACTACTTGTAAACCCATGGAACATCACTGAAGTTGCTTACTCGATAGGCTAT

GCGTTGAATATGCCTCCGGATGAAAGAGAAAAGAGACACAGGCATAACTTTGCTCATGTC

ACCACCCACACTGCTCAAGATTGGGCTGAAACTTTTGTAAGTGAACTTAACGATACTGTC

TTTGAAGCTCAGCTTAGGACAAGACAGGTCCCACCTTTACTTCCAACAAGAATTGCTATG

GAACGTTATTTTCAGTCCAAGAATCGATTGCTTATTCTGGGTTTCAATGCTACGTTGACT

GAACAAGTGGAGTCTCCTGGAAGGAGAGGTGGTGATCAGATTAAAGAGATGGAGCTTAAA

TTGCATCCAGAACTGAAACATCCCTTGAGCATACTTTGTAATGATCCAAAGACGACTATT

GTTGTCCTAAGTGGAAGTGACAGAAGTGTCCTGGATGATAACTTTGGAGAGTACAATATG

TGGTTGGCTGCAGAGAATGGGATGTTTTTACGTCTAACAACTGGAGAATGGATGACAACA

ATGCCAGAGCACCTAAACATGGATTGGGTTGAGAGCGTAAAGCATGTCTTTGAGTACTTC

ACCGAAAGAACACCTAGATCCCATTTTGAACACCGTGAGACTTCACTTTTGTGGAACTAT

AAGTATGCTGATGCTGAATTCGGAAGGCTTCAAGCAAGAGATATGTTGCAGCACTTGTGG

ACTGGACCGATTTCTAATGCTGCTGTAGATGTTGTCCAAGGCAGCCGATCTGTTGAGGTC

CGCTCTGTTGGTGTTACCAAGGGTGCAGCAATTGATCGTATCTTAGGGGAAATAGTTCAT

AGCCAATCTATGATTACACCAATCGATTATGTTTTATGCATCGGCCACTTTCTTGGAAAG

GATGAAGATATTTATACATTTTTTGAGCCAGAGCTTCCTGCTGAGCCTGTTGGCATTAGA

ACGAAGGCAGCTGAGATAATCAAGCCATCTATGGAGCGTAAAGTCTCCTCTAAGCTGTCA

AACATGAAGAACAACTCGAGGACAACTCAAGGTAAAGCACAGAAGGTCTCAACTGGAGCT

GACAGGAAAACTACTAGTAACTGCACTGTAACGGGATCTCGGTCACCACACGAAAACATC

TCATGTCATGAAGGATCATCTGTTCTGGACCTCAAAGGAGAGAATTACTTCTCGTGCGCA

GTAGGTCGGAAGTGTTCAAATGCCCGTTATCTGCTCGACACGTCTGATGATGTGGTCACA

TTTGTCAAGGAGCTAGCAGAGTCGTGTGGC

>Isoform0025377 pos:930-3239

TGCATCCCTGTGTTTCTCGATGAAGAAATAGTTCATCAATATTACAATGGTTACTGCAAC

AATGTATTGTGGCCATTATTTCACTATCTTGGGCTTCCGCAAGAGGATCGTTTGGCAACT

ACCCGTAGTTTCCAATCCCAGTTTGATGCTTACAAGCGTGCAAACCAAATGTTTGCTGAC

GTGGTGAACCAACACTATGAAGAGGGAGATGTGGTTTGGTGCCATGACTACCATCTTATG

TACCTCCCAAAATGCCTTAAAGAGTATAACAGGCAAATGAAAGTTGGATGGTTCCTTCAT

ACACCCTTTCCCTCCTCCGAAATTTACAGGACATTGCCATCACGATCAGAGTTGTTGAGA

TCTGTTCTTGCTGCTGATTTGGTCGGATTTCACACTTACGATTATGCAAGGCATTTTGTT

AGTGCCTGTACGCGGATTCTGGGATTTGAAGGCACCCCTGAAGGGGTGGAGGATCAAGGA

AGGTTGACTCGAGTTGCTGCATTTCCTATTGGGATAGACTCTGACCGTTTCACTCAAGCA

CTTGAGCTTCCAAAAGTTAAAGATCACATGAAAGATTTAAGAGAGAGATTTGCTGGCCGA

AAGGTGATGTTAGGTGTTGACCGACTCGACATGATTAAAGGAATACCTCAGAAAATTTTG

GCATTCGAAAAGTTCCTTGAGGAAAATCCTTCTTGGTGTGATAAAGTAGTTCTATTGCAA

ATAGCTGTGCCAACTAGAACAGATGTTCCTGAATATCAAAAACTTACCAGCCAGGTCCAT

GAAATTGTTGGACGCATTAATGGCCGTTTTGGAACACTCTCTGCAGTTCCTATACATCAT

CTGGATCGGTCTCTTGACTTCAATGCATTATGTGCACTCTATGCAGTCACTGATGTGGCT

CTTGTAACATCACTGAGGGATGGGATGAATCTAGTAAGCTATGAATTTGTGGCTTGCCAA

GAGCAAAAGAAGGGGGTTCTCATTCTCAGTGAATTTGCAGGAGCTGCACAATCTCTGGGT

GCTGGAGCACTACTTGTAAACCCATGGAACATCACTGAAGTTGCTTACTCGATAGGCTAT

GCGTTGAATATGCCTCCGGATGAAAGAGAAAAGAGACACAGGCATAACTTTGCTCATGTC

ACCACCCACACTGCTCAAGATTGGGCTGAAACTTTTGTAAGTGAACTTAACGATACTGTC

TTTGAAGCTCAGCTTAGGACAAGACAGGTCCCACCTTTACTTCCAACAAGAATTGCTATG

GAACGTTATTTTCAGTCCAAGAATCGATTGCTTATTCTGGGTTTCAATGCTACGTTGACT

GAACAAGTGGAGTCTCCTGGAAGGAGAGGTGGTGATCAGATTAAAGAGATGGAGCTTAAA

TTGCATCCAGAACTGAAACATCCCTTGAGCATACTTTGTAATGATCCAAAGACGACTATT

GTTGTCCTAAGTGGAAGTGACAGAAGTGTCCTGGATGATAACTTTGGAGAGTACAATATG

TGGTTGGCTGCAGAGAATGGGATGTTTTTACGTCTAACAACTGGAGAATGGATGACAACA

ATGCCAGAGCACCTAAACATGGATTGGGTTGAGAGCGTAAAGCATGTCTTTGAGTACTTC

ACCGAAAGAACACCTAGATCCCATTTTGAACACCGTGAGACTTCACTTTTGTGGAACTAT

AAGTATGCTGATGCTGAATTCGGAAGGCTTCAAGCAAGAGATATGTTGCAGCACTTGTGG

ACTGGACCGATTTCTAATGCTGCTGTAGATGTTGTCCAAGGCAGCCGATCTGTTGAGGTC

CGCTCTGTTGGTGTTACCAAGGGTGCAGCAATTGATCGTATCTTAGGGGAAATAGTTCAT

AGCCAATCTATGATTACACCAATCGATTATGTTTTATGCATCGGCCACTTTCTTGGAAAG

GATGAAGATATTTATACATTTTTTGAGCCAGAGCTTCCTGCTGAGCCTGTTGGCATTAGA

ACGAAGGCAGCTGAGATAATCAAGCCATCTATGGAGCGTAAAGTCTCCTCTAAGCTGTCA

AACATGAAGAACAACTCGAGGACAACTCAAGGTAAAGCACAGAAGGTCTCAACTGGAGCT

GACAGGAAAACTACTAGTAACTGCACTGTAACGGGATCTCGGTCACCACACGAAAACATC

TCATGTCATGAAGGATCATCTGTTCTGGACCTCAAAGGAGAGAATTACTTCTCGTGCGCA

GTAGGTCGGAAGTGTTCAAATGCCCGTTATCTGCTCGACACGTCTGATGATGTGGTCACA

TTTGTCAAGGAGCTAGCAGAGTCGTGTGGC

>Isoform0025274 pos:918-3227

TGCATCCCTGTGTTTCTCGATGAAGAAATAGTTCATCAATATTACAATGGTTACTGCAAC

AATGTATTGTGGCCATTATTTCACTATCTTGGGCTTCCGCAAGAGGATCGTTTGGCAACT

ACCCGTAGTTTCCAATCCCAGTTTGATGCTTACAAGCGTGCAAACCAAATGTTTGCTGAC

GTGGTGAACCAACACTATGAAGAGGGAGATGTGGTTTGGTGCCATGACTACCATCTTATG

TACCTCCCAAAATGCCTTAAAGAGTATAACAGGCAAATGAAAGTTGGATGGTTCCTTCAT

ACACCCTTTCCCTCCTCCGAAATTTACAGGACATTGCCATCACGATCAGAGTTGTTGAGA

TCTGTTCTTGCTGCTGATTTGGTCGGATTTCACACTTACGATTATGCAAGGCATTTTGTT

AGTGCCTGTACGCGGATTCTGGGATTTGAAGGCACCCCTGAAGGGGTGGAGGATCAAGGA

AGGTTGACTCGAGTTGCTGCATTTCCTATTGGGATAGACTCTGACCGTTTCACTCAAGCA

CTTGAGCTTCCAAAAGTTAAAGATCACATGAAAGATTTAAGAGAGAGATTTGCTGGCCGA

AAGGTGATGTTAGGTGTTGACCGACTCGACATGATTAAAGGAATACCTCAGAAAATTTTG

GCATTCGAAAAGTTCCTTGAGGAAAATCCTTCTTGGTGTGATAAAGTAGTTCTATTGCAA

ATAGCTGTGCCAACTAGAACAGATGTTCCTGAATATCAAAAACTTACCAGCCAGGTCCAT

GAAATTGTTGGACGCATTAATGGCCGTTTTGGAACACTCTCTGCAGTTCCTATACATCAT

CTGGATCGGTCTCTTGACTTCAATGCATTATGTGCACTCTATGCAGTCACTGATGTGGCT

CTTGTAACATCACTGAGGGATGGGATGAATCTAGTAAGCTATGAATTTGTGGCTTGCCAA

GAGCAAAAGAAGGGGGTTCTCATTCTCAGTGAATTTGCAGGAGCTGCACAATCTCTGGGT

GCTGGAGCACTACTTGTAAACCCATGGAACATCACTGAAGTTGCTTACTCGATAGGCTAT

GCGTTGAATATGCCTCCGGATGAAAGAGAAAAGAGACACAGGCATAACTTTGCTCATGTC

ACCACCCACACTGCTCAAGATTGGGCTGAAACTTTTGTAAGTGAACTTAACGATACTGTC

TTTGAAGCTCAGCTTAGGACAAGACAGGTCCCACCTTTACTTCCAACAAGAATTGCTATG

GAACGTTATTTTCAGTCCAAGAATCGATTGCTTATTCTGGGTTTCAATGCTACGTTGACT

GAACAAGTGGAGTCTCCTGGAAGGAGAGGTGGTGATCAGATTAAAGAGATGGAGCTTAAA

TTGCATCCAGAACTGAAACATCCCTTGAGCATACTTTGTAATGATCCAAAGACGACTATT

GTTGTCCTAAGTGGAAGTGACAGAAGTGTCCTGGATGATAACTTTGGAGAGTACAATATG

TGGTTGGCTGCAGAGAATGGGATGTTTTTACGTCTAACAACTGGAGAATGGATGACAACA

ATGCCAGAGCACCTAAACATGGATTGGGTTGAGAGCGTAAAGCATGTCTTTGAGTACTTC

ACCGAAAGAACACCTAGATCCCATTTTGAACACCGTGAGACTTCACTTTTGTGGAACTAT

AAGTATGCTGATGCTGAATTCGGAAGGCTTCAAGCAAGAGATATGTTGCAGCACTTGTGG

ACTGGACCGATTTCTAATGCTGCTGTAGATGTTGTCCAAGGCAGCCGATCTGTTGAGGTC

CGCTCTGTTGGTGTTACCAAGGGTGCAGCAATTGATCGTATCTTAGGGGAAATAGTTCAT

AGCCAATCTATGATTACACCAATCGATTATGTTTTATGCATCGGCCACTTTCTTGGAAAG

GATGAAGATATTTATACATTTTTTGAGCCAGAGCTTCCTGCTGAGCCTGTTGGCATTAGA

ACGAAGGCAGCTGAGATAATCAAGCCATCTATGGAGCGTAAAGTCTCCTCTAAGCTGTCA

AACATGAAGAACAACTCGAGGACAACTCAAGGTAAAGCACAGAAGGTCTCAACTGGAGCT

GACAGGAAAACTACTAGTAACTGCACTGTAACGGGATCTCGGTCACCACACGAAAACATC

TCATGTCATGAAGGATCATCTGTTCTGGACCTCAAAGGAGAGAATTACTTCTCGTGCGCA

GTAGGTCGGAAGTGTTCAAATGCCCGTTATCTGCTCGACACGTCTGATGATGTGGTCACA

TTTGTCAAGGAGCTAGCAGAGTCGTGTGGC

>Isoform0003236 pos:66-2375

TGCATCCCTGTGTTTCTCGATGAAGAAATAGTTCATCAATATTACAATGGTTACTGCAAC

AATGTATTGTGGCCATTATTTCACTATCTTGGGCTTCCGCAAGAGGATCGTTTGGCAACT

ACCCGTAGTTTCCAATCCCAGTTTGATGCTTACAAGCGTGCAAACCAAATGTTTGCTGAC

GTGGTGAACCAACACTATGAAGAGGGAGATGTGGTTTGGTGCCATGACTACCATCTTATG

TACCTCCCAAAATGCCTTAAAGAGTATAACAGGCAAATGAAAGTTGGATGGTTCCTTCAT

ACACCCTTTCCCTCCTCCGAAATTTACAGGACATTGCCATCACGATCAGAGTTGTTGAGA

TCTGTTCTTGCTGCTGATTTGGTCGGATTTCACACTTACGATTATGCAAGGCATTTTGTT

AGTGCCTGTACGCGGATTCTGGGATTTGAAGGCACCCCTGAAGGGGTGGAGGATCAAGGA

AGGTTGACTCGAGTTGCTGCATTTCCTATTGGGATAGACTCTGACCGTTTCACTCAAGCA

CTTGAGCTTCCAAAAGTTAAAGATCACATGAAAGATTTAAGAGAGAGATTTGCTGGCCGA

AAGGTGATGTTAGGTGTTGACCGACTCGACATGATTAAAGGAATACCTCAGAAAATTTTG

GCATTCGAAAAGTTCCTTGAGGAAAATCCTTCTTGGTGTGATAAAGTAGTTCTATTGCAA

ATAGCTGTGCCAACTAGAACAGATGTTCCTGAATATCAAAAACTTACCAGCCAGGTCCAT

GAAATTGTTGGACGCATTAATGGCCGTTTTGGAACACTCTCTGCAGTTCCTATACATCAT

CTGGATCGGTCTCTTGACTTCAATGCATTATGTGCACTCTATGCAGTCACTGATGTGGCT

CTTGTAACATCACTGAGGGATGGGATGAATCTAGTAAGCTATGAATTTGTGGCTTGCCAA

GAGCAAAAGAAGGGGGTTCTCATTCTCAGTGAATTTGCAGGAGCTGCACAATCTCTGGGT

GCTGGAGCACTACTTGTAAACCCATGGAACATCACTGAAGTTGCTTACTCGATAGGCTAT

GCGTTGAATATGCCTCCGGATGAAAGAGAAAAGAGACACAGGCATAACTTTGCTCATGTC

ACCACCCACACTGCTCAAGATTGGGCTGAAACTTTTGTAAGTGAACTTAACGATACTGTC

TTTGAAGCTCAGCTTAGGACAAGACAGGTCCCACCTTTACTTCCAACAAGAATTGCTATG

GAACGTTATTTTCAGTCCAAGAATCGATTGCTTATTCTGGGTTTCAATGCTACGTTGACT

GAACAAGTGGAGTCTCCTGGAAGGAGAGGTGGTGATCAGATTAAAGAGATGGAGCTTAAA

TTGCATCCAGAACTGAAACATCCCTTGAGCATACTTTGTAATGATCCAAAGACGACTATT

GTTGTCCTAAGTGGAAGTGACAGAAGTGTCCTGGATGATAACTTTGGAGAGTACAATATG

TGGTTGGCTGCAGAGAATGGGATGTTTTTACGTCTAACAACTGGAGAATGGATGACAACA

ATGCCAGAGCACCTAAACATGGATTGGGTTGAGAGCGTAAAGCATGTCTTTGAGTACTTC

ACCGAAAGAACACCTAGATCCCATTTTGAACACCGTGAGACTTCACTTTTGTGGAACTAT

AAGTATGCTGATGCTGAATTCGGAAGGCTTCAAGCAAGAGATATGTTGCAGCACTTGTGG

ACTGGACCGATTTCTAATGCTGCTGTAGATGTTGTCCAAGGCAGCCGATCTGTTGAGGTC

CGCTCTGTTGGTGTTACCAAGGGTGCAGCAATTGATCGTATCTTAGGGGAAATAGTTCAT

AGCCAATCTATGATTACACCAATCGATTATGTTTTATGCATCGGCCACTTTCTTGGAAAG

GATGAAGATATTTATACATTTTTTGAGCCAGAGCTTCCTGCTGAGCCTGTTGGCATTAGA

ACGAAGGCAGCTGAGATAATCAAGCCATCTATGGAGCGTAAAGTCTCCTCTAAGCTGTCA

AACATGAAGAACAACTCGAGGACAACTCAAGGTAAAGCACAGAAGGTCTCAACTGGAGCT

GACAGGAAAACTACTAGTAACTGCACTGTAACGGGATCTCGGTCACCACACGAAAACATC

TCATGTCATGAAGGATCATCTGTTCTGGACCTCAAAGGAGAGAATTACTTCTCGTGCGCA

GTAGGTCGGAAGTGTTCAAATGCCCGTTATCTGCTCGACACGTCTGATGATGTGGTCACA

TTTGTCAAGGAGCTAGCAGAGTCGTGTGGC
